# Supplementary material for: Online computer or therapist-guided cognitive behavioral therapy in university students with anxiety and/or depression: study protocol of a randomised controlled trial
Source: BMJ Open. 2021 Nov 26;11(11):e049554. doi: 10.1136/bmjopen-2021-049554 (PMC8628330; doi:10.1136/bmjopen-2021-049554)
Supplement: Supplementary data [file bmjopen-2021-049554supp002.pdf]

UNIVERSITEIT VAN AMSTERDAM

Appendix B

Subject information for participation in medical scientific research

UvAcare project

Examining the effectiveness of a web-based intervention for symptoms of depression and/or anxiety among students and PhD students.

Introduction

Dear student, dear PhD student,

You are kindly asked to take part in a medical-scientific study. Participation is voluntary. Participation requires your written consent. You have received this letter because, based on the online survey that you have filled in, you appear to experience symptoms of depression and/or anxiety. This means that you might benefit from an online intervention. Before you decide whether you want to participate in this study, you will be given an explanation about what the study involves. Please read this information carefully and ask the research team for an explanation if you have any questions. You can also ask the independent expert (dhr. drs. Daan Tempelman (D.A.J.Tempelman@uva.nl, 0205254772) for additional information. You may also discuss it with your partner, classmates, friends, or family.

Additional information about participating in the study can be found in the enclosed 'Medical Research Brochure'.

1. General information

This study is conducted at the department of developmental psychology of the University of Amsterdam (UvA), in collaboration with the Student Medical Healthservice UvA-HvA and VU Amsterdam. The Medical Ethics Committee of the Academic Medical Center (AMC) has approved this study. General information about the assessment of research can be found in the 'Medical Research Brochure'.

2. Purpose of the study

First of all, the UvAcare project is part of a care project that aims to offer additional care to students and PhD students at the UvA in the coming years using different online interventions, which will be developed throughout the projectand subsequently conduct research to improve existing care and make it more accessible.

NL64929.018.18 – versie [5] [16-07-2019]

Therefore, this project will investigate the effectiveness of an online intervention for university students and PhD students with symptoms of depression and/or anxiety. The efficacy of a guided transdiagnostic intervention and an unguided, automatic, version of the same online intervention will be compared to the efficacy of treatment as usual. Treatment as usual consists of the option to seek help for example of a student psychologist, a general practitioner, or of a specialist in primary or secondary mental health care. In addition, the efficacy of the guided online intervention will be compared to the efficacy of the unguided online intervention. Principally, we will examine the reduction of depression and/or anxiety symptoms before and after treatment, and compared to no treatment at all.

3. Background of the study

The goal of the UvAcare project is to recognize mental health problems in college students and PhD students and to treat those in need at an early stage. It's important to tackle this early on because college and PhD years are considered to be a peak period for the first onset of common mental disorders, such as depression and anxiety. Preventive interventions may limit the development of symptoms and may prevent low (academic) achievement and academic drop out. Research has shown that guided web-based interventions are effective in treating symptoms of depression and anxiety in the general population. In this study we examine the effectiveness of a guided and unguided transdiagnostic treatment adapted to college students' needs. We would like to examine whether these transdiagnostic web-based interventions are effective in treating symptoms of depression and/or anxiety when compared to treatment as usual services for students and PhD students.

4. What participation involves

We kindly ask you to complete a couple of questionnaires: before the self-help online intervention, at 5 weeks, at 8 weeks, at 6 months and at 12 months. In addition we ask you to fill in a short questionnaire before each treatment session, or weekly in case of the treatment-as-usual condition (8 sessions/weeks) to monitor your mental health.

Research

If you decide that you would like to participate, you will receive an invitation for a clinical diagnostic interview administered through a phone call. The purpose of this interview is to determine whether you have a diagnosis of depression or anxiety and to check whether you are eligible to participate in this study. This interview lasts approximately 30-45 minutes. If you are eligible to participate we ask you to complete the first set of online questionnaires. After completing this, it will be determined by drawing lots, whether you will be assigned either to the guided intervention, to the unguided intervention or to treatment as usual. Your chance to be assigned to any of the groups is one third.

The online intervention

The online intervention consists of 7 sessions and one booster session 4 weeks after completing the last regular session. It is recommended to complete one session a week. One session takes about 45 to 60 minutes. The sessions consist of text, exercises and audio-visual components (e.g. videos). Participants in the unguided intervention receive automatic feedback messages and reminders, and there is a possibility for personal contact. In additon, participants in the guided intervention receive personal feedback on every session from their personal online coach.

Treatment as usual

In the treatment-as-usual condition you will receive information about existing care facilities inside and outside of the UvA. Based on this information, you will be encouraged to seek the help you might need choosing the type of care that most appeals to you.

5. What is expected of you

In order to carry out the study properly, it is important that you follow the study instructions.

The study instructions require that you:

- complete the online questionnaires at thirteen points in time: before the beginning of the online intervention, before each treatment session or weekly during the intervention (8 sessions/weeks in total), at 5 weeks, at 8 weeks, at 6 months and at 12 months after the start of the intervention,
- will be interviewed by phone four times before the beginning of the online intervention

Thus, we will contact you after the intervention for questionnaires (after the intervention, at 6 months and 12 months).

It is important that you contact the research team (uvacare@uva.nl):

- if you no longer want to participate in the study,
- if your contact details change.

6. Possible side effects/complications

We expect that participation in this study entails negligible risks.

Supplemental material BMJ Publishing Group Limited (BMJ) disclaims all liability and responsibility arising from any reliance placed on this supplemental material which has been supplied by the author(s) BMJ Open

7. Possible advantages and disadvantages

It is important that you consider the possible benefits and disadvantages before you decide to participate. An advantage of participating is the possibility of the guided or unguided online intervention. The intervention is 24 hours per day available. There is also the possibility of personal contact during this period. The intervention might have a positive effect on possible symptoms of depression and/or anxiety. Moreover, your participation may contribute to increased knowledge about the treatment and help us in determining its feasibility and efficacy for college students with symptoms of anxiety and/or depression. We expect that participation in this study does not entail any risks. However, it is possible that you may experience some levels of distress when participating in the diagnostic interview, filling out the questionnaires or working on the intervention, since elaborating on potential unpleasant emotions and working on your current problems might be confronting. In addition, participating requires some time and effort.

8. If you do not want to participate or you want to stop participating in the study

It is up to you to decide whether or not to participate in the study. Participation is voluntary. If you participate in the study, you can always change your mind and decide to stop, at any time during the study. Moreover, you do not have to say why you are stopping. The data collected until that time will still be used for the study. If there is any new information about the study that is important to you, the investigator will let you know. You will then be asked whether you still want to continue your participation.

9. End of the study

Your participation in the study stops when:

- You have completed all the questionnaires
- You choose to stop
- The investigator considers it best for you to stop
- The government or Medical Research Ethics Committee decides to stop the study.

The study is concluded once all the participants have completed all the questionnaires of the study.

After processing the data, the investigator will inform you about the most important results of the study.

Klein A, *et al. BMJ Open* 2021; 11:e049554. doi: 10.1136/bmjopen-2021-049554

10. Usage and storage of your data

Your personal data will be collected, used and stored for this study. This concerns data such as your name, address, date of birth and data about your health. The collection, use and storage of your data is required to answer the questions asked in this study and to publish the results. Your data will also be forwarded to the VU Amsterdam, governed by the same European guidelines for personal data protection. This, however, includes solely data that are essential for the study. We ask your permission for the use of your data.

Confidentiality of your data

To protect your privacy, any personal information that could identify you will be stored separately from your research data and will be given a code. Your name and other information that can directly identify you will be omitted. Data can only be traced back to you with the encryption key. The encryption key remains safely stored in the local research institute. The data that is sent to the sponsor will only contain the code, not your name or other data with which you can be identified. The data cannot be traced back to you in reports and publications about the study.

Access to your data for verification

Some people can access all your data at the research location. Including the data without a code. This is necessary to check whether the study is being conducted in a good and reliable manner. Persons who have access to your data for review are members of the research team who monitor the study, the principal investigators, and national and international supervisory authorities, for example, the Healthcare and Youth Inspectorate. They will keep your data confidential. The members of the research team will only trace back to an individual in the case of an emergency, e.g. suicidal ideation. In that case the research team will contact the participant and provide information on further help. It is therefore possible that the research team will contact you during the intervention. However, this is only the case if you consider hurting yourself and seem to be in need of extra help. We ask you to consent to this access.

Retention period of your data

Your data must be kept for 15 years at the research location.

Storage and use of data for other research

Your data may also be of importance for other scientific research in the field of web-based intervention in treating symptoms of depression and/or anxiety. To this end, your data will be stored for 15 years. You can indicate on the consent form whether or not you agree with this. If you do not agree with this, you can still participate in the current study.

Withdrawing consent

You can withdraw your consent to the use of your personal data at any time. This applies to this study and also to storage and use for future research. The study data collected until the moment you withdraw your consent will still be used in the study.

More information about your rights when processing data

For general information about your rights when processing your personal data, you can consult the website of the Dutch Data Protection Authority.

If you have questions about your rights, please contact the person responsible for the processing of your personal data. For this study, that is:

Dhr. Prof Dr. Reinout Wiers, University of Amsterdam. See Appendix A for contact details.

If you have questions or complaints about the processing of your personal data, we advise you to first contact the research location. You can also contact the Data Protection Officer of the UvA (please see contact details in Appendix A) or the Dutch Data Protection Authority.

Registration of the study

Information about this study is included in a list of medical-scientific studies namely CCMO [NL64929.018.18/ToetsingOnline]. It does not contain any information that can be traced to you. After the study, the website may display a summary of the results of this study.

11. Compensation for participation

The internet-based intervention used in the study is free of charge to you. Participants who will complete the follow-up assessments will be offered a voucher worth of 10 euros. Additionally, amongst every 100 of these students, a tablet will be granted. If you stop before the study is over, you will not receive a compensation for participation.

12. Any questions?

If you have any questions, please contact the **study team** through email or phone (uvacare @uva.nl, secretary 020-5256830). If you would like any independent advice about participation in this study, you may contact dhr. drs. Daan Tempelman (D.A.J.Tempelman@uva.nl, 020-5254772). He knows about the study but is not involved in it.

If you have any complaints, you may contact the member of the Ethics Review Board (ERB) of the Faculty of Social and Behavioural Sciences (FMG) of the University of Amsterdam: dhr. dr. Wery van den Wildenberg, W.P.M.vandenWildenberg@uva.nl, 020-5256686. All the relevant details can be found in **Appendix A**: Contact details.

UNIVERSITEIT VAN AMSTERDAM

13. Signing the consent form

If you've had enough time to consider and you would like to participate in the study, we will ask you to confirm this in writing on the appended consent form. By your written permission you indicate that you have understood the information and consent to participation in the study. There are three ways to give your written consent:

- Print the consent form, check all relevant boxes, sign the form and send it via post to the following address (no stamp required):

  UvA-FMG
  Ontwikkelingspsychologie
  t.a.v. UvAcare (code 1041)
  Antwoordnummer 3521
  1000 RA Amsterdam

- If you're not able to print and/or send the consent form you can use the link in the invitation e-mail to leave your address details. We will send an envelope with a printed form and a retour envelope. You can easily sign your consent and return the form via post.
- If you rather sign and give the form directly to us there are informed consent forms at the Secretaries Office of Developmental Psychology, that you can sign directly.

Any questions about the consent form will be answered by the research team. The signature sheet is kept by the researcher.

Thank you for your attention.

Kind regards,

Mr. Dr. R. Wiers, professor Developmental Psychology
Ms. Dr. A. Klein, Senior Researcher Developmental Psychology
Mr. P. Vonk, director General Practitioners Practice UvA-HvA
Ms. C. van der Heijde, Senior Researcher General Practitioners Practice UvA-HvA

14. Appendices to this information

A. Contact details
B. Informed Consent Form
C. 'Medical Research Brochure: General information for Subjects'

Appendix A: contact details

Principal Investigators:

Mr. Prof. Reinout Wiers,
Professor Developmental Psychology

Ms. Dr. Anke Klein,
Senior Researcher Developmental Psychology
University of Amsterdam, Faculty of Behavioural and Movement Sciences
Programme group Developmental Psychology
Postbus 15916, 1001 NK Amsterdam

Secretary: Mrs. Helma Kruijk | E: W.G.S.M.Kruijk@uva.nl | T: 0205256830

Mr. Peter Vonk, MD,
Director General Practitioners Practice UvA-HvA,

Ms. Dr. Claudia van der Heijde,
Senior Researcher General Practitioners Practice UvA-HvA
University of Amsterdam,
Department of Research, development and prevention
Oude Turfmarkt 151, 1012 GC Amsterdam

Secretary: Mrs. Ester van Run | E: E.M.M.vanRun@uva.nl | T: 0205252857

Research team email: uvacare@uva.nl

Independent expert:

Dhr. Drs. Daan Tempelman
General practitioner,
Student Medical Healthservice UvA-HvA
University of Amsterdam
Oude Turfmarkt 151
1012 GC Amsterdam
E: D.A.J.Tempelman@uva.nl | T: 0205254772

Complaints contact:

Dhr. Dr. Wery van den Wildenberg
Faculty of Behavioural and Movement Sciences
Programme group Developmental Psychology
Postbus 15916, 1001 NK Amsterdam
E: W.P.M.vandenWildenberg@uva.nl | T: 0205256686

Klein A, *et al. BMJ Open* 2021; 11:e049554. doi: 10.1136/bmjopen-2021-049554

UNIVERSITEIT VAN AMSTERDAM

Data Protection Officer:
Mw. mr. drs. J.M.C. Krol
Spui 21, Kamernummer: 1.18
1012 WX Amsterdam E:
fg@uva.nl

UNIVERSITEIT VAN AMSTERDAM

Appendix B: Informed consent participant

UvAcare project

Title: *Examining the effectiveness of a web-based intervention for symptoms of depression and/or anxiety in university students and PhD students.*

- I have read the subject information form. I was also able to ask questions. My questions have been answered to my satisfaction. I had enough time to decide whether to participate.
- I know that participation is voluntary. I know that I may decide at any time not to participate after all or to withdraw from the study. I do not need to give a reason for this.
- I know that some people may have access to all my data to verify the study. These people are listed in this information sheet. I consent to the inspection by them.
- I can give consent at the start of the telephonic diagnostic interviews to record the phone call. If I do not agree with this, I can still participate in this study.
- I agree to my data being forwarded to other universities within the European Union, governed by European guidelines for personal data protection. This must, however, be essential for the study. The data must be shared in encoded form without stating my name.
- I **do** ☐
  do no☐
  consent to keeping my personal data longer and to use it for future research in the field of web- based intervention in treating symptoms of depression and/or anxiety.
- I **do** ☐
  do no☐
  consent to being contacted again after this study for a follow-up study
- I want to participate in this study.

Name participant:
Email address participant:
Signature: Date : ___/___/ _____

I hereby declare that I have fully informed this study subject about this study.

If information comes to light during the course of the study that could affect the study subject's consent, I will inform him/her of this in a timely fashion.

Name of investigator (or his/her representative):
Signature: Date: / / _

NL64929.018.18 – versie [5] [16-07-2019]
