# Supplementary material for: Online computer or therapist-guided cognitive behavioral therapy in university students with anxiety and/or depression: study protocol of a randomised controlled trial
Source: BMJ Open. 2021 Nov 26;11(11):e049554. doi: 10.1136/bmjopen-2021-049554 (PMC8628330; doi:10.1136/bmjopen-2021-049554)
Supplement: Supplementary data [file bmjopen-2021-049554supp003.pdf]

Appendix C

Example of automated feedback in the computer-guided condition:

After completing for example session six, all participants receive the same message in the messaging function; "*Dear participant, Congratulations on finishing the sixth session. We know the training is not always easy, so well done. In this session you were once again reminded that practice makes perfect. Keep trying to find the motivation you had when you started and use the tips from all the sessions thus far to work on difficult situations and challenges. You are now starting with the second to last session of the training module. We wish you once again good luck with the next session! Kind regards, the research team*".

Example of feedback in the therapist-guided condition:

An example of feedback on the sixth session; "*Dear [name of participant], Thank you for sending in the exercises from the sixth and second-to-last session. It's great that you have been persistent with this training over the past weeks! You started this session by taking stock of how your challenges from last week went. It is nice to read that you have faced your first challenge. You have taken a first step to actively conquer your fears, well done! Have you thought about rewarding yourself? This week you detected a safety behavior: carrying items to prepare for the worst-case scenarios despite never having to use them. It's great you could identify it, since you can only start making changes once you have become aware of these behaviours! In this session you have chosen to stick with the challenging area you picked last time, which was dealing with situations where there is no clear process like a queue in a busy cafe. It's great that you made a plan to challenge yourself in the coming week! You might benefit from making sure the link is clear between the activities you planned and the challenging area you chose to focus on. The more concrete and specific your plan is, the*

easier it will be to carry out. It is also important to have a detailed plan so you can accurately judge the difficulty of each situation. This way, you can start with easier challenges and build your way up to harder ones. To follow the examples from the previous session, your plan could be something like "Monday: order coffee with a friend (easier), Wednesday: go alone to the doctor's office where there is a clear system for the queue (moderate), Saturday: order coffee on my own (harder)". It is also possible to start with very small tasks: sometimes just imagining the situation can be a useful first step. Don't forget that every attempt is worth being rewarded and deserves respect! Plan something nice or fun for yourself as soon as you have confronted a situation. This week you also completed the optional module on rest and relaxation. I hope these strategies were useful for you and that you can use them going forward. I am also glad to see that you are continuing to plan positive activities to incorporate into your daily life, like going for a walk, getting takeout, or visiting your friends! In the seventh (and for now last) session of this training you will have the opportunity to draw up a plan for the future. You will get to plan exercises for the coming weeks, and we will look back on the training and your successes. I am looking forward to hearing back from you then! Kind regards, [name of coach]".
