# Supplementary material for: Online computer or therapist-guided cognitive behavioral therapy in university students with anxiety and/or depression: study protocol of a randomised controlled trial
Source: BMJ Open. 2021 Nov 26;11(11):e049554. doi: 10.1136/bmjopen-2021-049554 (PMC8628330; doi:10.1136/bmjopen-2021-049554)
Supplement: Author's manuscript [file bmjopen-2021-049554.draft_revisions.pdf]

BMJ Open

BMJ Open is committed to open peer review. As part of this commitment we make the peer review history of every article we publish publicly available.

When an article is published we post the peer reviewers' comments and the authors' responses online. We also post the versions of the paper that were used during peer review. These are the versions that the peer review comments apply to.

The versions of the paper that follow are the versions that were submitted during the peer review process. They are not the versions of record or the final published versions. They should not be cited or distributed as the published version of this manuscript.

BMJ Open is an open access journal and the full, final, typeset and author-corrected version of record of the manuscript is available on our site with no access controls, subscription charges or pay-per-view fees (http://bmjopen.bmj.com).

If you have any questions on BMJ Open's open peer review process please email info.bmjopen@bmj.com

BMJ Open

Online Cognitive Behavioral Therapy with Therapist versus Automated Feedback in University Students with Anxiety and/or Depression: Study Protocol of a Randomized Controlled Trial

Journal:	*BMJ Open*
Manuscript ID	bmjopen-2021-049554
Article Type:	Protocol
Date Submitted by the Author:	27-Jan-2021
Complete List of Authors:	Klein, Anke; University of Amsterdam Psychology,<br>Wolters, N. E.; University of Amsterdam, Developmental Psychology<br>Bol, E. J. M.; University of Amsterdam, Developmental Psychology<br>Koelen, J.; University of Amsterdam, Developmental Psychology<br>de Koning, L.; University of Amsterdam, Developmental Psychology<br>Roetink, S. S. M.; University of Amsterdam, Developmental Psychology<br>van Blom, J. J.; University of Amsterdam, Developmental Psychology<br>Pronk, T.; University of Amsterdam Psychology<br>van der Heijde, Claudia; Universiteit van Amsterdam<br>Salemink, Elske; Utrecht University<br>Bolinski, Felix; Vrije Universiteit Amsterdam, Clinical, Neuro- & Developmental Psychology<br>Riper, Heleen; Vrije Universiteit Amsterdam<br>Karyotaki, Eirini ; Vrije Universiteit Amsterdam, Clinical Neuro and Developmental Psychology<br>Cuijpers, Pim; Vrije Universiteit Amsterdam, Department of Clinical Psychology<br>Schneider, S.; Ruhr University Bochum<br>Rapee, Ronald M.; Macquarie University, Psychology<br>Vonk, Peter ; Universiteit van Amsterdam<br>Wiers, Reinout; University of Amsterdam, Developmental Psychology
Keywords:	Adult psychiatry < PSYCHIATRY, Anxiety disorders < PSYCHIATRY, Depression & mood disorders < PSYCHIATRY

SCHOLARONE™
Manuscripts

I, the Submitting Author has the right to grant and does grant on behalf of all authors of the Work (as defined in the below author licence), an exclusive licence and/or a non-exclusive licence for contributions from authors who are: i) UK Crown employees; ii) where BMJ has agreed a CC-BY licence shall apply, and/or iii) in accordance with the terms applicable for US Federal Government officers or employees acting as part of their official duties; on a worldwide, perpetual, irrevocable, royalty-free basis to BMJ Publishing Group Ltd ("BMJ") its licensees and where the relevant Journal is co-owned by BMJ to the co-owners of the Journal, to publish the Work in this journal and any other BMJ products and to exploit all rights, as set out in our licence.

The Submitting Author accepts and understands that any supply made under these terms is made by BMJ to the Submitting Author unless you are acting as an employee on behalf of your employer or a postgraduate student of an affiliated institution which is paying any applicable article publishing charge ("APC") for Open Access articles. Where the Submitting Author wishes to make the Work available on an Open Access basis (and intends to pay the relevant APC), the terms of reuse of such Open Access shall be governed by a Creative Commons licence – details of these licences and which Creative Commons licence will apply to this Work are set out in our licence referred to above.

Other than as permitted in any relevant BMJ Author's Self Archiving Policies, I confirm this Work has not been accepted for publication elsewhere, is not being considered for publication elsewhere and does not duplicate material already published. I confirm all authors consent to publication of this Work and authorise the granting of this licence.

For peer review only

Online Cognitive Behavioral Therapy with Therapist versus Automated Feedback in University Students with Anxiety and/or Depression: Study Protocol of a Randomized Controlled Trial

A. M. Klein[12*], N. E. Wolters[1], E. J. M. Bol[1], J. Koelen[1], L. de Koning[1], S. S. M. Roetink[1], J. J. van Blom[1], T. Pronk[13], C. M. van der Heijde[2], E. Salemink[1], F. Bolinksi[4], H. Riper[4], E. Karyotaki[4], P. Cuijpers[4], S. Schneider[5], R. M. Rapee[6], P. Vonk[2], & R. W. Wiers[1]

1. Addiction Development and Psychopathology (ADAPT)-lab, Developmental Psychology, Department of Psychology, University of Amsterdam
2. Department of Research, Development and Prevention, Student Health Service, University of Amsterdam, Amsterdam, The Netherlands
3. Behavioural Science Lab, Faculty of Social and Behavioural Sciences, University of Amsterdam, Amsterdam, The Netherlands
4. Department of Clinical Neuro- and Developmental Psychology, Amsterdam Public Health Research Institute, Vrije Universiteit Amsterdam, The Netherlands
5. Clinical Child and Adolescent Psychology, Ruhr University Bochum, Germany
6. Centre for Emotional Health, Macquarie University, Australia

* Corresponding authors: A.M. Klein, Ph.D., a.m.klein@uva.nl; Reinout Wiers, Ph.D., r.w.h.j.wiers@uva.nl

For peer review only

Abstract

Introduction: Emerging adulthood is a phase in life that is associated with an increased risk to develop a variety of mental health disorders including anxiety and depression. However, less than 25% of university students receive professional help for their mental health complaints. Internet-based Cognitive Behavioral Therapy (iCBT) may entail useful interventions in a format that is attractive for university students. The aim of this study protocol is to test the effectiveness of a therapist-guided versus a computer-guided transdiagnostic iCBT program with a main focus on anxiety and depression.

Methods and Analysis: University students with anxiety and/or depressive symptoms will be randomized to a 1) 7-week iCBT program (excluding booster-session) with therapist feedback, 2) the same iCBT program with computer feedback only or, 3) care as usual. Primary outcome variables are self-reported levels of anxiety as measured with the General Anxiety Disorder-7 (GAD-7), and self-reported levels of depression as measured with the Patient Health Questionnaire-9 (PHQ-9). Secondary outcomes include treatment adherence, client satisfaction, medical service use, substance use, quality of life and academic achievement. Assessments will take place at baseline (t1), mid-intervention (t2), post-intervention at 8 weeks after randomization (t3), at 6 months (t4) and 12 months (t5) post-randomization. Social anxiety and perfectionism are included as potentially important predictors of treatment outcome. Power calculations are based on a 3 (group) x 3 (measurement: pre- mid- and post intervention) interaction resulting in an aimed sample of 276 participants. Data will be analyzed based on intention-to-treat, and per protocol samples using mixed linear models.

Ethics and Dissemination: The current study was approved by the METC of the Academic Medical Center, Amsterdam, The Netherlands (number: NL64929.018.18). Results of this trial will be published in peer-reviewed journals.

For peer review only - http://bmjopen.bmj.com/site/about/guidelines.xhtml

Trial registration: Dutch Trial register number: NL7328.

(287 words)

Keywords: Anxiety, Depression, eHealth, online Cognitive Behavioral Treatment (ICBT), Therapist-guided versus computer-guided, Randomized controlled trial, University students

For peer review only

Article Summary

Strength and limitations of this study

- The aim of the current study is to further improve online mental health care in university students with anxiety and depression.
- An online transdiagnostic CBT program with therapist guidance versus a computer-based guidance will be compared to each other and to care as usual
- The procedure of the trial is developed to make it easily accessible and anonymous, and the full procedure is online.
- The therapists are not fully blinded to the treatment condition.
- The post-treatment outcomes rely on self-reports of the participants.

For peer review only

Background

Emerging adulthood is a crucial stage in life that includes the transition from the teenage years to full-fledged adulthood (18-25 years). It is a phase during which many young adults attend university to receive education and training to obtain a degree that will be the basis for their adult working life [1]. It is also the phase that is associated with an increased risk of developing a variety of mental health disorders, such as anxiety and depression [2;3;4] and comorbidity is the rule rather than the exception [5]. Recent studies found that between 16-46% of all students suffer from mental health disorders [6;2]. Moreover, these problems have been associated with poorer academic performance, academic dropout, decreased labor market functioning, and poorer social and health outcomes [7;6;8;9;10]. Early detection and effective intervention are thus crucial with regard to the long-term prognosis of mental disorders, especially as untreated mental disorders have a mean duration between 4 and 23 years [11].

Online treatment programs may be useful as an intervention in a format that is particularly attractive for university students, given the fact that less than 25% of students with mental health problems utilize available face to face services [12]. University students might avoid help due to several reasons including stigmatization, lack of perceived urgency, preference to deal with it themselves, threat to identity formation and goal setting, and/or skepticism regarding treatment effectiveness [13; 14] This group may be more likely to seek help if an effective, more flexible, easily accessible, and anonymous intervention strategy is provided. A scoping review by [15] concluded that internet therapies have (untapped) potential to provide better information about mental health and may improve the cost efficacy of mental health services. Moreover, their study indicated that young people, in general, prefer eHealth treatments relative to face-to-face services.

For peer review only - http://bmjopen.bmj.com/site/about/guidelines.xhtml

Several studies demonstrated that the effectiveness of guided internet-based Cognitive Behavioral Therapy (iCBT) programs for anxiety and depression are comparable to conventional (face-to-face) CBT [for meta-analyses 16; 17]There is also some evidence for the effectiveness of internet interventions for young adults and university student populations with various mental health problems, including depression, anxiety, eating disorders, stress, alcohol, and sleep problems [e.g.,18; 19; 20; 21; 22]. For example, a recent meta-analysis by [20] focused on university students with mental problems who self-selected themselves for a psychological online intervention (guided / unguided). They found small intervention effects for depression (g = 0.18, 95% confidence interval [CL; 0.08, 0.27]) and anxiety (g = 0.27, 95% confidence interval [CL; 0.13, 0.40] in university students. It should be noted, however, that the effects on anxiety were not significant any more after adjusting for publication bias. [20] concluded that clearly more research is needed to explore ways to increase treatment effectiveness and to study predictors of treatment outcome.

The current study includes a group of university students and focuses on two of the most common mental disorders in this group, namely anxiety and depression [2] As comorbidity is high, we will use a transdiagnostic iCBT intervention which has the advantage to target both disorders at the same time and thereby might increase treatment effectiveness [for a systemic review and meta-analysis see 23]. Moreover, iCBT programs can be provided with or without additional personal guidance. Guidance usually implies written or verbal feedback by the therapist to the patient's homework assignments, and other forms of (safe) communication (e.g., chat, telephone) within an online environment with varying degrees of interactive features [25]. Both approaches (with and without personal guidance) have shown to be efficacious, although programs with (therapist) guidance generally report a higher effect size [for meta-analyses, 24; 17], even though it should be noted that a recent meta-analysis by [20] found no difference between personal versus without personal guidance. To date, little is

For peer review only - http://bmjopen.bmj.com/site/about/guidelines.xhtml

known about the effectiveness for therapist-guided versus a computer-guided version of the same intervention tested *within* the same study when applied to a university student sample size with mental health concerns [see also 26;20;27]. This could have important health economic implications considering the additional cost of human support. Therefore, the current study tests this transdiagnostic iCBT for anxiety and depression in three groups; a therapist-guided online self-help version, a computer-guided online version of the same program and care as usual. Primary outcomes were anxiety and depression, secondary measures were included to evaluate treatment adherence, academic achievement, medical service use and treatment satisfaction.

Internet-based programs, either therapist-guided with personal feedback or computer-guided with automated feedback, might suit some individuals better than others. It is important to focus on possible treatment predictors (e.g., including baseline symptoms severity, gender, personality factors and comorbidity), as this may help us understand more about the heterogeneity of treatment response [e.g. 20;28]. Specially, [20] found limited effectiveness on anxiety in university students in their meta-analysis. The current study will focus on the possible role of social anxiety and perfectionism, as there is some evidence that adults and children with social anxiety might benefit less from general CBT programs [for meta-analyses see 29; 30]. Likewise, self-critical perfectionism is a transdiagnostic personality factor that is generally associated with worse treatment outcome, including CBT [31]. Importantly, individuals with self-critical perfectionism have poorer social relationships, which in turn negatively impacts therapeutic progress [32].

Based on previous studies in the general population [17], we hypothesize that the computer-guided iCBT condition and computer-guided iCBT condition are significantly more effective than care-as-usual. In addition, we expect that the therapist-guided condition is significantly more effective than the computer-guided CBT condition.

For peer review only - http://bmjopen.bmj.com/site/about/guidelines.xhtml

Methods

Study design

The current study includes a Randomized Controlled Trial (RCT) design with three arms, consisting of (1) a therapist-guided transdiagnostic iCBT program with personalized feedback, (2) a computer-guided version of the same CBT program with automated feedback, and (3) Care-As-Usual (CAU). We used the SPIRIT reporting guidelines for this study [33].

Eligibility criteria

All student scoring 16 or higher on the Center for Epidemiological Studies Depression Scale (CES-D) [34] and/or a score of 5 or higher on the General Anxiety Disorder-7 (GAD-7) [35] were included in the study. There is no higher end cut off. Participants are excluded from the study if they 1) are younger than 16 years of age, 2) currently receive psychological treatment for anxiety and/or depression, 3) do not have access to a stable internet connection for the next twelve months, 4) if they meet the diagnostic criteria for (recent or current) psychotic disorder according to the Mini International Neuropsychiatric Interview (M.I.N.I) [35], 5) if they are at active high risk for suicide based on the MINI, and 6) if they do not give informed consent. Participants meeting exclusion criteria 4 and/or 5 are referred to local health care facilities.

Recruitment

All students enrolled at the University of Amsterdam (UvA), The Netherlands, receive an invitation via email that are sent centrally from the study platform. In addition, study advisors and study counselors of the UvA are informed about the study and are asked to refer

For peer review only - http://bmjopen.bmj.com/site/about/guidelines.xhtml

students who are interested in participating in the study to the research team so that they can be invited by email.

The invitation email contains general information about the study and a unique link to fill out the screening questionnaire. Participants who click on the invitation link are referred to the project platform. Here, they find an information letter and are asked to give online informed consent to the research team. Next, participants are asked to create an account after which they are directed to the screening questionnaire. Following the original invitation, two reminder emails are be sent one- and two weeks after the first invitation. As the study runs for three academic years (2019-2021), participants are asked at the beginning of the screening if they consent to being invited again later. Moreover, participants have the option to opt out of the study at any time, and they can also indicate that they do not want to receive any emails again.

Recruitment for the intervention phase of the study is based on the scores of the screening questionnaires (GAD-7 and CES-D). Participants who score above the cut-off on either anxiety and/or depressive symptoms receive an email with an online information brochure and informed consent document [see also: 35; 34; see Appendix A]. After informed consent is given by the participant, participants who fulfill all inclusion criteria are called by a trained clinical research assistant. A telephonic diagnostic interview [M.I.N.I.; 36] are administered to establish diagnoses with respect to mood and anxiety disorders, bipolar disorder, psychosis, and suicidal ideation.

Randomization, blinding and treatment allocation

Participants are randomly assigned to either the therapist-guided online intervention, the computer-guided online intervention or care as usual (control) condition (1:1:1 allocation ratio) directly following the baseline measurement. Randomization is stratified by gender and anxiety and /or depressive symptoms (based on the cutoff scores on the screening

For peer review only - http://bmjopen.bmj.com/site/about/guidelines.xhtml

questionnaire). Sequence generation is based on computer-generated random numbers and are allocated by an automatic system.

Due to the nature of the intervention, participants and researchers cannot be completely blinded to the assigned treatment condition, though note that the measurements in the intervention are blinded. Researchers are aware of the participants 'condition because they provide participants with feedback in the therapist-guided condition. In the computer-guided condition feedback is automatically generated by the system, but therapists do send reminders to this group as part of the intervention.

Intervention

The online transdiagnostic intervention focuses on anxiety and depression that is used in this study, 'ICare Prevent', was originally developed by [37] for the general German-speaking population, and translated and adapted by [38] and [39] for a Dutch undergraduate student population into Dutch and English. The intervention is provided to the participants through the eHealth platform Minddistrict. This platform enables researchers and healthcare providers to provide digital therapy to their participants and patients. On this platform, e-coaches can create a personal account for each participant, monitor the progress of the participants, provide support for the participants in the therapist-guided treatment condition, and respond to questions from the participants with the messaging function. Data processing and storage are in accordance with the ISO 27000 and NEN 7510 norms. A data processing agreement that complies with the GDPR has been signed between e-health provider Minddistrict and the University of Amsterdam.

The intervention is based on basic cognitive behavioral therapy principles for anxiety and depression with components of psychoeducation and motivational interviewing, including online exercises and home assignments. The intervention consists of 7 regular

For peer review only - http://bmjopen.bmj.com/site/about/guidelines.xhtml

sessions (45 - 60 minutes per session) and 1 booster session (4 weeks after the completion of the last session). From the second session onward, participants are able to follow eight additional optional modules based on their personal needs, including sleep, perfectionism, alcohol use, reduce rumination, self-worth, acceptance, appreciations, and rest and relaxation. In session five and six, users decide to follow either content directed at changing negative cognitions, or exposure to fear situations. They can decide to choose one additional module per session, and they are free to repeat this module or choose other modules in later sessions. Participants are free to decide when they would like to start a treatment session and if they want to do an additional module, but it is advised to do at least one and no more than two treatment sessions per week. For a full description of the intervention, see [39].

During the intervention, all participants receive online support in the form of messages or notifications in the messaging function. In both conditions, participants receive up to three weekly reminders via the messaging function when they are inactive. Moreover, participants in both conditions can use the messaging function to ask questions. All participants can ask technical or usability related questions. During the trial, training and weekly supervision for the e-coaches who are guiding the participants are provided by a licensed mental health care psychologist. Participants in the computer-guided eHealth condition receive automatically generated feedback messages (see Appendix B for an example). In contrast, in the therapist-guided condition, the e-coaches (trained psychologists on minimal BA level) provide detailed feedback based on participants' output of the sessions, and this feedback is displayed at the bottom of the session they completed (see Appendix B for an example). The coach spends approximately 30 minutes on providing feedback per session and intends to give feedback within 5 working days after the session is completed by the participant.

For peer review only - http://bmjopen.bmj.com/site/about/guidelines.xhtml

Care as usual

Participants in the care as usual condition are informed and referred to conventional care services and encouraged to seek the help they need. Medical services used by the participants during the RCT are monitored through self-report questions at t3, t4 and t5. Participants in the care as usual condition are assessed at the same time points as the two intervention conditions including the weekly questionnaires during the first 7 weeks.

Suicide risk monitoring

All participants receive an information brochure with all relevant contact information as part of the information package they receive prior to the study. In addition, the brief version of the PHQ-9, the PHQ-4 [40] is administered before each treatment session, or weekly via email (care as usual). Suicide risk is monitored with the help of a suicide risk protocol that was created specifically for this study [Klein et al., 2018. *Suicide protocol*]. Suicide risk is monitored using item 3 ('feeling down, depressed, or hopeless') of the PHQ-4 and item 9 ('thought that you would be better off dead, or of hurting yourself') of the Beck Depression Inventory (BDI)-II scale [41]. If deemed necessary, participants are referred to appropriate services, including their GP or 113zelfmoordpreventie (online platform for suicide prevention). In addition, the participant also receives a pop-up message on the questionnaire page with relevant contact information right after filling in the questionnaire (i.e., where they can find help if needed with relevant contact information).

Assessments

The RCT includes five assessment points: (1) a baseline assessment including a diagnostic interview and questionnaires, (2) a mid-intervention questionnaire, 5 weeks after randomization, (3) a post-treatment questionnaire, 8 weeks after randomization and (4) follow-

For peer review only - http://bmjopen.bmj.com/site/about/guidelines.xhtml

up measurements, 6 months, and (5) 12 months after randomization (see also Figure 1). In addition, prior to each treatment session, or weekly in the CAU-condition, participants fill out a short online questionnaire. This questionnaire briefly assesses symptom severity and monitors suicidal ideation.

Primary outcomes

The GAD-7 [35] and the Patient Health Questionnaire-9 (PHQ-9) [42] are the primary outcomes in the study and assess respectively *anxiety and depressive symptoms* on a continuous scale. The 7-item GAD is a self-report questionnaire measuring anxiety symptoms. All items are scored on a scale from 0 (not at all') to 3 'nearly every day'), with total scores ranging from 0 to 21. A higher score on the questionnaire is associated with a more severe experience of anxiety symptoms. The GAD-7 questionnaire has good psychometric properties including a good test re-test reliability (ICC = .83) [35] and a good internal consistency ($.79 < \alpha < 0.91$) [43]. The PHQ-9 questionnaire is used to screen for self-reported symptoms of depression. The 9 items are scored on a 0-3 scale, resulting in a total score between 0 and 27. A higher score on the scale indicates a more severe experience of depressive symptoms. The PHQ-9 is a valid and reliable tool with good sensitivity (.88), and high specificity (.94) [44; 45] (see Table 1 for an overview of all questionnaires on all time points).

Secondary outcomes

Alcohol and *drug use* are measured with respectively the Alcohol Use Disorders Identification Test-Concise (AUDIT-C) [46; 47] and the Drug Abuse Screening Test – 10 items (DAST-10) [48]. The AUDIT-C is a brief screening tool for identifying early and/or heavy alcohol use. The scale consists of 3 items, all scored on a 0-4 scale, resulting in a total score ranging from 0-12. The DAST-10 consists of 10 items. Item responses are 'yes' or 'no'.

For peer review only - http://bmjopen.bmj.com/site/about/guidelines.xhtml

Higher scores indicate a more severe level of drug abuse problems. The DAST-10 reports high validity (α = .94) and reliability in a broad range of settings and populations [49].

Quality of life is measured with the EuroQol-5D-5L scale (EQ-5D-5L) [50] The EuroQol-5D-5L scale assesses health-related wellbeing on five dimensions: mobility, self-care, ordinary activities, discomfort, and mood state. The sixth item of the EQ-5D-5L is a VAS scale, ranging from 0-100, asking the participant to rate their health perception at that moment. The EQ-5D-5L has high levels of acceptability and sensitivity [51; 52].

Academic achievement is measured with objective and subjective measures. Students are asked to report their number of European Credit Transfer System (ECTS) as an objective measure of their academic performance. Additionally, the work impairment subscale of the Presenteeism Scale for Students (PSS-WIS) [53] is administered to assess subjective academic achievement on a 5-point Likert scale ranging from 'always' (0) to 'never' (5). The PSS-WIS is a valid (ICC=.88 [95% CI, $p < .001$]) and reliable (test-re-test reliability $r = .80$, $p < .001$) self-report measure to screen for impaired work performance due to (mental) health problems [53].

Medical service use is measured with two items from the Treatment Inventory of Costs in Patients with mental disorders (TiC-P) [54]. The first item includes the frequency of contact with conventional care services (e.g. general practitioner, study advisor, psychologist, medical specialist). The second item includes the use of medication during the length of the treatment period.

Client satisfaction with treatment is measured with the Client Satisfaction Questionnaire – 8 items (CSQ-8) [55; 56]. The CSQ-8 consists of 8 items scored on a 1 ('quite dissatisfied')-4 ('very satisfied') scale, with total scores ranging between 8-32. The CSQ-8 is a standardized satisfaction measure reporting very good internal consistency (α=.83-.93) and high validity [57].

For peer review only - http://bmjopen.bmj.com/site/about/guidelines.xhtml

Finally, following previous studies [for a systematic review, 58] *treatment adherence* is measured by tracking the activities in Minddistrict. We collect the total number of modules completed, time spent per module and the number of times the participants log into the Minddistrict platform.

Predictors of treatment outcome

Social anxiety is measured with the Social Interaction Anxiety Scale-6 item (SIAS-6) [59] and the brief Mini-Social Phobia Inventory (Mini-SPIN) [60]. The brief Mini-Social Phobia Inventory (Mini-SPIN) [60] is included to screen for generalized social anxiety disorder. The scale shows strong sensitivity (93.8%) and good internal consistency (α = 0.85) [61; 62]. Besides the administration on T1-T5, the Mini-SPIN is administered before each treatment session, or weekly via email (care as usual), to monitor the social anxiety status of the participant.

Perfectionism is measured with the short version of the Self-Critical Perfectionism subscale of the Depressive Experiences Questionnaire (DEQ-SCP) [63]. The subscale consists of 12 items. The DEQ-SCP scale is a simple, valid scale demonstrating good psychometric properties, in both clinical and student samples [64].

Besides the inclusion of social anxiety and perfectionism, we include several variables to study possible predictors of treatment outcome including demographic variables (i.e. age, gender, study direction, faculty, national/international student), baseline severity, personality factors and comorbidity for explorative analyses.

Statistical analyses

Sample size calculation. The power analysis is based on the mixed factors 3x3 interaction difference between the three treatment arms and three time points (pre- mid- and

For peer review only - http://bmjopen.bmj.com/site/about/guidelines.xhtml

post measurement). We anticipate a small interaction effect (Cohen's f = .1) at post-treatment based on a recent meta-analysis that focused on the effectiveness of therapist-guided versus computer-guided psychotherapy in treating depression and anxiety [17]. We determined a statistical power (1-ß) of .8 and corrected alpha of $\alpha = .05$. With a two-tailed hypothesis, we need 204 participants per intervention group to be able to detect a statistically significant result. Previous literature has shown that web-based interventions have a relatively high reported range of drop-out in university students, varying between approximately 30% - 40%post treatment [e.g. 20] Calculating with a dropout rate of 35%, the minimum required total sample for the RCT is $N_{total} = 276$ participants.

Clinical analyses. Mixed Linear Models analyses will be conducted with SPSS and R. To get a more realistic picture of the true efficacy of treatment and completer analyses will also be performed for those participants who adhere to the protocol.

Predictor analyses. Predictors of treatment outcome will be investigated on an exploratory basis with pre-defined predictors: social anxiety and perfectionism, baseline symptoms severity, gender, personality factors and comorbidity). Analyses will be conducted using regression analyses, with interaction effects and structural equation modelling.

Patient and public involvement

A focus group of university students helped with the design of the study. This group also helped with writing the consent letters, the website texts and checked the clearness of all instructions in the online platform. All university students of the UvA will receive an email with the (anonymized) results at the group level when the project is finished.

Discussion

For peer review only - http://bmjopen.bmj.com/site/about/guidelines.xhtml

Even though many university students face mental problems, only relatively few students utilize available services [12]. Internet Cognitive Behavioral Therapy (iCBT) programs are easily accessible, flexible, and relatively anonymous [25]. Hence, they might be a good alternative for reaching out to this group [e.g., 14]. The current study aims to compare the effectiveness of online interventions for elevated levels of anxiety and/or depression in university students enrolled at the University of Amsterdam: a therapist-guided transdiagnostic CBT program, a computer-guided version of the same program, and care as usual. Several moderators are included that may potentially amplify or attenuate treatment response, and so may provide new knowledge on effectiveness for different subgroups.

Strengths of this study are the use of a transdiagnostic iCBT program, its large sample size, and the generalizability of the findings since exclusion criteria are minimal (e.g., there are almost no exclusion criteria concerning co-morbid disorders; we created a Dutch and English version so that both national and international student can participate). Moreover, the current CBT program is flexible, because participants can complete sessions at their own pace and it includes separate modules focusing on related problems (e.g., stress, sleep, alcohol use). This flexibility has both strengths and limitations. On the one hand, participants will not feel pressured, they can work on a session whenever they have time do to so, and they have freedom in choosing additional modules that fit their situation. On the other hand, this might result in a different therapeutic process for each participant, which makes it more difficult to compare the different groups. Also, this flexibility makes it more difficult to investigate the effective components of the program. One way to investigate this is to follow the course of the symptoms by analyzing the pre-session questionnaire. Some participants might find it difficult to stay motivated or decide between the different treatment module options. Therefore, one of the limitations of this study is a potentially high degree of drop-out, especially in the computer-guided program and in the care as usual condition [24].

For peer review only - http://bmjopen.bmj.com/site/about/guidelines.xhtml

Consequently, the therapist is not able to provide personal feedback directly following each session, which might decrease treatment alliance. To minimize drop-out, participants receive up to three weekly reminders via the messaging function when they are inactive. In addition, we provide all participants with technical support and suicide risk monitoring. Also, there may be a potential confound of "feedback length", as the feedback in the therapist-guided condition is textually longer than the automated feedback in the computer-guided condition. Due to the large sample size but limited human resources, and our priority to maintain the privacy of participants, all measurements are self-reported, and may therefore be more subjective than clinician-rated reports. Finally, our automated study design allows for blinded assessments throughout the intervention, which on the downside may lead to some assessments not being aligned with the intervention phase (e.g., "mid-treatment" questionnaires being completed before session 4 has been reached.)

In sum, the current trial is unique as it compares two types of the same transdiagnostic iCBT intervention; a therapist-guided, versus a computer-guided version in a large sample that includes a one-year follow-up. This study will therefore improve our understanding of the need of individualized feedback for computer-based-interventions. The findings will significantly add to knowledge on the treatment of depression and anxiety in university students and will give insight into treatment predictors that may determine why some students benefit and others do not.

Declarations

Ethical approval, consent to participate and dissemination

The current study was approved by the METC of the Academic Medical Center, Amsterdam, The Netherlands (number: NL64929.018.18), and follows all Dutch ethical legislations. Any unintended effects will be reported to the METC committee. All procedures are in

For peer review only

accordance with the latest version of the Helsinki declaration, and comply to the GDPR rules (2018). All protocol changes will be reported to the ethical committees, and trial participants per email. Active informed consent from participants is asked for the entire procedure. Results of this trial will be published in peer-reviewed journals.

Data protection, data access and data management

The current project fulfils all GDPR requirements, data was recruited in The Netherlands. All authors have access to the data. A data management plan was developed and approved by an external data privacy officer of the University of Amsterdam. Data and the data-management plan will be shared upon request by contacting the corresponding author.

Competing interests

The authors state that they have no conflict of interest.

Funding

This work was supported by a grant from the University of Amsterdam (Spui 21, 1012GC, +31205251400) awarded to Dr. A. M. Klein (Co-PI), Drs. Peter Vonk (PI; p.vonk@uva.nl; Oude Turfmarkt 151, 1012LA, Amsterdam, University of Amsterdam), Claudia van der Heijde (Co-PI) and Prof. Reinout Wiers (PI; r.w.h.j.wiers@uva.nl; Nieuwe achtergracht 129, 1001NK, Amsterdam, University of Amsterdam). A steering group from the University of Amsterdam monitors the processes of the entire trial and approves mid-term evaluation.

Author Contributions

RW (PI), PV (PI), AK (Co-PI) and CvdH (Co-PI) received funding for the project. All authors contributed to the design of the study. AK drafted the main part of the manuscript,

For peer review only - http://bmjopen.bmj.com/site/about/guidelines.xhtml

NW and LdK assisted in writing parts of the manuscript under supervision of AK. All authors provided feedback on earlier versions of the manuscript. AK, SR and JvB coordinate the data recruitment and data collection. All authors approved the final version of the manuscript for submission.

Acknowledgements

We would like to thank the student psychologists, the student deans, colleagues of academic affairs, the colleagues from the technical support department (TOP), the student advisory boards who were involved in the focus group from the University of Amsterdam and M. van der Hoff and R. Atteveld for their help with the design of the project.

Trial registration and Trial Status

Dutch Trial register number: NL7328. Registered 10 October 2018, https://www.trialregister.nl/trial/7328. The current protocol is the first version. The starting date of the recruitment is 31st January 2019 and runs until March 2022. Dr. A. M. Klein is the contact person for both public and scientific queries: uvacare@uva.nl, +205252878, Oude Turfmarkt 151, 1012LA Amsterdam.

For peer review only

References

1 Arnett JJ. Emerging adulthood: A theory of development from the late teens through the twenties. *Am Psychol*2000;55(5):469.

2 Auerbach RP, Mortier P, Bruffaerts R, et al. WHO World Mental Health Surveys International College Student Project: Prevalence and distribution of mental disorders. *Journal of abnormal psychology*2018;127(7):623.

3 De Girolamo G, Dagani J, Purcell R, et al. Age of onset of mental disorders and use ofmental health services: needs, opportunities and obstacles. *Epidemiol Psychiatr Sci*2012;1;21(1):47.

4 Zivin K, Eisenberg D, Gollust SE, et al. Persistence of mental health problems and needs in a college student population. *J Affect Disord*2009;117(3):180-5.

5 Auerbach RP, Mortier P, Bruffaerts R, Alonso J, Benjet C, Cuijpers P, Demyttenaere K, Ebert DD, Green JG, Hasking P, Lee S. Mental disorder comorbidity and suicidal thoughts and behaviors in the World Health Organization world mental health surveys international college student initiative. *Int J Methods Psychiatr Res*2019;28(2):e1752.

6 Auerbach RP, Alonso J, Axinn WG, et al. Mental disorders among college students in the World Health Organization world mental health surveys. *Psychol Med*2016;46(14):2955-70.

7 Alonso J, Mortier P, Auerbach RP, et al Severe role impairment associated with mental disorders: results of the WHO world mental health surveys international college student project. *Depress Anxiety*2018;35(9):802-14.

8 Bolinski F, Boumparis N, Kleiboer A, et al. The effect of e-mental health interventions on academic performance in university and college students: A meta-analysis of randomized controlled trials. *Internet Interv*2020:100321.

9 McGorry PD, Purcell R, Goldstone S, et al. Age of onset and timing of treatment for mental and substance use disorders: implications for preventive intervention strategies and models of care. *Curr Opin Psychiatry*2011;24(4):301-6.

10 Kessler RC, Berglund PA, Bruce ML, et al. The prevalence and correlates of untreated serious mental illness. *Health Serv Res*2001;36(6 Pt 1):987.

11 Wang PS, Berglund P, Olfson M, et al. Failure and delay in initial treatment contact after first onset of mental disorders in the National Comorbidity Survey Replication. *Arch Gen Psychiatry*2005;62(6):603-13.

For peer review only - http://bmjopen.bmj.com/site/about/guidelines.xhtml

12 Buchanan JL. Prevention of depression in the college student population: a review of the literature. *Arch Psychiatr Nurs*2012;26(1):21-42.

13 Eisenberg D, Hunt J, Speer N, et al. Mental health service utilization among college students in the United States. *J Nerv Ment Dis*2011;199(5):301-8.

14 Van der Heijde CM, Vonk P, Meijman FJ. Self-regulation for the promotion of student health. Traffic lights: the development of a tailored web-based instrument providing immediate personalized feedback. *Health Psychol Behav Med*2015;3(1):169-89.

15 Boydell KM, Hodgins M, Pignatiello A, et al. Using technology to deliver mental health services to children and youth: a scoping review. *J Can Acad Child Adolesc Psychiatry*2014;23(2):87.

16 Carlbring P, Andersson G, Cuijpers P, et al. Internet-based vs. face-to-face cognitive behavior therapy for psychiatric and somatic disorders: an updated systematic review and meta-analysis. *Cogn Behav Ther*2018;47(1):1-8.

17 Cuijpers P, Noma H, Karyotaki E, et al. Individual, group, telephone, self-help and internet-based cognitive behavior therapy for adult depression: A network meta-analysis of delivery methods. *JAMA Psychiatry*2019;76:700-7.

18 Davies EB, Morriss R, Glazebrook C. Computer-delivered and web-based interventions to improve depression, anxiety, and psychological well-being of university students: a systematic review and meta-analysis. *J Med Internet Res*2014;16(5):e130.

19 Ebert DD, Zarski AC, Christensen H, et al. Internet and computer-based cognitive behavioral therapy for anxiety and depression in youth: a meta-analysis of randomized controlled outcome trials. *PloS One*2015;10(3):e0119895.

20 Harrer M, Adam SH, Baumeister H, et al. Internet interventions for mental health in university students: A systematic review and meta-analysis. *Int J Methods Psychiatr Res*2019;28(2):e1759.

For peer review only

21 Sethi S, Campbell AJ, Ellis LA. The use of computerized self-help packages to treat adolescent depression and anxiety. *J Technol Hum Serv*2010;28(3):144-60.

22 Riper H, van Straten A, Keuken M, et al. Curbing problem drinking with personalized-feedback interventions: a meta-analysis. *Am J Prev Med*2009;36(3):247-55.

23 Newby JM, McKinnon A, Kuyken W, et al. Systematic review and meta-analysis of transdiagnostic psychological treatments for anxiety and depressive disorders in adulthood. *Clin Psychol Rev*2015;40:91-110.

24 Richards D, Richardson T. Computer-based psychological treatments for depression: a systematic review and meta-analysis. *Clin Psychol Rev*2012;32(4):329-42.

25 Andersson G, Cuijpers P, Carlbring P, et al. Guided Internet-based vs. face-to-face cognitive behavior therapy for psychiatric and somatic disorders: a systematic review and meta-analysis. *World Psychiatry*2014;13(3):288-95.

26 Berger T, Hämmerli K, Gubser N, et al. Internet-based treatment of depression: a randomized controlled trial comparing guided with unguided self-help. *Cog Behav Ther*2011;40(4):251-66.

27 Renfrew ME, Morton DP, Morton JK, et al. The Influence of Three Modes of Human Support on Attrition and Adherence to a Web-and Mobile App–Based Mental Health Promotion Intervention in a Nonclinical Cohort: Randomized Comparative Study. *J Med Internet Res*2020;22(9):e19945.

28 Kessler RC, Van Loo HM, Wardenaar KJ, et al. Using patient self-reports to study heterogeneity of treatment effects in major depressive disorder. *Epidemiol Psychiatr Sci*2017;26(1):22-36.

29. Hudson JL, Rapee RM, Lyneham HJ et al. Comparing outcomes for children with different anxiety disorders following cognitive behavioural therapy. Behav Res Ther2015;72:30-37.

For peer review only

30. Norton PJ, & Price, EC. A meta-analytic review of adult cognitive-behavioral treatment outcome across the anxiety disorders. J Nerv Ment Dis2007;195(6):521-31.

31 Löw CA, Schauenburg H, Dinger U. Self-criticism and psychotherapy outcome: A systematic review and meta-analysis. *Clin Psychol Rev*2020;75:101808.

32 Shahar G, Blatt SJ, Zuroff DC, et al. Perfectionism impedes social relations and response to brief treatment for depression. *J Soc Clin Psychol*2004;23(2):140-54.

33 Chan A-W, Tetzlaff JM, Gøtzsche PC, et al. SPIRIT 2013 Explanation and Elaboration: Guidance for protocols of clinical trials. *BMJ Open*2013;346:e7586

34 Beekman AT, Deeg DJ, Van Limbeek J, et al. Brief communication.: criterion validity of the Center for Epidemiologic Studies Depression scale (CES-D): results from a community-based sample of older subjects in the Netherlands. *Psychol Med*1997;27(1):231-5.

35 Spitzer RL, Kroenke K, Williams JB, et al. A brief measure for assessing generalized anxiety disorder: the GAD-7. *Arch Intern Med*2006;166(10):1092-7.

36 Lecrubier Y, Sheehan DV, Weiller E, et al. The Mini International Neuropsychiatric Interview (MINI). A short diagnostic structured interview: reliability and validity according to the CIDI. *Eur Psychiatry*1997;12(5):224-31.

37 Weisel KK, Zarski AC, Berger T, et al. Transdiagnostic tailored internet-and mobile-based guided treatment for major depressive disorder and comorbid anxiety: study protocol of a randomized controlled trial. *Front Psychiatry*2018;9:274.

38 Bolinski F, Kleiboer A, Karyotaki E, et al. Effectiveness of a transdiagnostic individually tailored Internet-based and mobile-supported intervention for the indicated prevention of depression and anxiety (ICare Prevent) in Dutch college students: study protocol for a randomised controlled trial. *Trials*2018;19(1):118.

For peer review only - http://bmjopen.bmj.com/site/about/guidelines.xhtml

39 Karyotaki E, Klein AM, Riper H, et al. Examining the effectiveness of a web-based intervention for symptoms of depression and anxiety in college students: study protocol of a randomised controlled trial. *BMJ Open*2019;9(5):e028739.

40 Kroenke K, Spitzer RL, Williams JB, et al. An ultra-brief screening scale for anxiety and depression: the PHQ–4. *Psychosomatics*2009;50(6):613-21.

41 Beck AT, Steer RA, Ball R, et al. Comparison of Beck Depression Inventories-IA and-II in psychiatric outpatients. *J Pers Assess*1996;67(3):588-97.

42 Kroenke K, Spitzer RL. The PHQ-9: a new depression diagnostic and severity measure. *Psychiatric Ann*2002;32(9):509-15.

43 Dear BF, Titov N, Sunderland M, et al. Psychometric comparison of the generalized anxiety disorder scale-7 and the Penn State Worry Questionnaire for measuring response during treatment of generalised anxiety disorder. *Cogn Behav Ther*2011;40(3):216-27.

44 Wittkampf KA, Naeije L, Schene AH, et al. Diagnostic accuracy of the mood module of the Patient Health Questionnaire: a systematic review. *Gen Hosp Psychiatry*2007;29(5):388-95.

45 Levis B, Benedetti A, Thombs BD. Accuracy of Patient Health Questionnaire-9 (PHQ-9) for screening to detect major depression: individual participant data meta-analysis. *BMJ Open*2019;365.

46 Bush K, Kivlahan DR, McDonell MB, et al. The AUDIT alcohol consumption questions (AUDIT-C): an effective brief screening test for problem drinking. *Arch Intern Med*1998;158(16):1789-95.

47 Verhoog S, Dopmeijer JM, de Jonge JM, et al. The use of the alcohol use disorders identification test–consumption as an indicator of hazardous alcohol use among university students. *Eur Addict Res*2020;26(1):1-9.

48 Skinner HA. The drug abuse screening test. *Addict Behav*1982;7(4):363-71.

For peer review only - http://bmjopen.bmj.com/site/about/guidelines.xhtml

49 Maisto SA, Carey MP, Carey KB, et al. Use of the AUDIT and the DAST-10 to identify alcohol and drug use disorders among adults with a severe and persistent mental illness. *Psychol Assess*2000;12(2):186.

50 Group TE. EuroQol-a new facility for the measurement of health-related quality of life. *Health Policy*1990;16(3):199-208.

51 Heslin M, Chua KC, Trevillion K, et al. Psychometric properties of the five-level EuroQoL-5 dimension and Short Form-6 dimension measures of health-related quality of life in a population of pregnant women with depression. *BJPsych Open*2019;5(6).

52 König HH, Heider D, Lehnert T, et al. Health status of the advanced elderly in six European countries: results from a representative survey using EQ-5D and SF-12. *Health Qual Life Outcomes*2010;8(1):143.

53 Matsushita M, Adachi H, Arakida M, et al. Presenteeism in college students: reliability and validity of the Presenteeism Scale for Students. *Qual Life Res*2011;20(3):439-46.

54 Bouwmans C, De Jong K, Timman R, et al. Feasibility, reliability and validity of a questionnaire on healthcare consumption and productivity loss in patients with a psychiatric disorder (TiC-P). *BMC Health Ser Res*2013;13(1):217.

55 Larsen DL, Attkisson CC, Hargreaves WA, et al. Assessment of client/patient satisfaction: development of a general scale. *Eval Program Plann*1979;2(3):197-207.

56 De Brey H. A cross-national validation of the client satisfaction questionnaire: the Dutch experience. *Eval Program Plann*1983;6(3-4):395-400.

57 Attkisson CC, Greenfield TK. *Client Satisfaction Questionnaire-8 and Service Satisfaction Scale-30*. In: Maruish ME, eds. *The Use of Psychological Testing for Treatment Planning and Outcome Assessment*. (pp 402–420). Lawrence Erlbaum Associates, Inc.

58 Christensen H, Griffiths KM, Farrer L. Adherence in internet interventions for anxiety and depression: systematic review. *J Med Internet Res*2009;11(2):e13.

For peer review only - http://bmjopen.bmj.com/site/about/guidelines.xhtml

59 Peters L, Sunderland M, Andrews G, et al. Development of a short form Social Interaction Anxiety (SIAS) and Social Phobia Scale (SPS) using nonparametric item response theory: The SIAS-6 and the SPS-6. *Psychol Assess*2012 Mar;24(1):66.

60 Connor KM, Kobak KA, Churchill LE, et al. Mini-SPIN: A brief screening assessment for generalized social anxiety disorder. *Depress Anxiety*2001;14(2):137-40.

61 Weeks JW, Spokas ME, Heimberg RG. Psychometric evaluation of the mini-social phobia inventory (Mini-SPIN) in a treatment-seeking sample. *Depress Anxiety*2007;24(6):382-91.

62 Seeley-Wait E, Abbott MJ, Rapee RM. Psychometric properties of the mini-social phobia inventory. *Prim Care Companion J Clin Psychiatry*2009;11(5):231.

63 Blatt SJ, D'Afflitti JP, Quinlan DM. Experiences of depression in normal young adults. *J Abnorm Psychol*1976 Aug;85(4):383.

64 Desmet M, Vanheule S, Groenvynck H, et al. The depressive experiences questionnaire. *Eur J Psychol Assess*2007;23(2):89-98.

For peer review only

Table 1. *Overview of the measures separately for each assessment point*

	Questionnaire	Assessment points					
		Baseline (t1)	Pre-sessions	Mid-intervention (t2)	Post-intervention (t3)	6 months follow-up (t4)	12 months follow-up (t5)
Anxiety	GAD-7	X	X*	X	X	X	X
Depression	PHQ-9	X	X*	X	X	X	X
Social anxiety	SIAS-6	X		X	X	X	X
Social anxiety	Mini-SPIN	X	X	X	X	X	X
Alcohol use	AUDIT-C	X		X	X	X	X
Drug use	DAST-10	X		X	X	X	X
Quality of life	EQ-5D	X			X	X	X
Objective academic achievement	**	X			X	X	X
Subjective academic achievement	PSS-WIS	X			X	X	X
Perfectionism	DEQ-SCP (short)	X					
Suicide risk	BDI-II (item 9) PHQ-4 (item 3)		X				
Medical Service Use	TiC-P				X	X	X
Satisfaction with intervention	CSQ-8				X		

For peer review only - http://bmjopen.bmj.com/site/about/guidelines.xhtml

*PHQ-4, brief anxiety and depression measure to monitor symptoms. **Self-developed questionnaire. GAD-7 = General Anxiety Disorder-7 items, PHQ-9 = Patient Health Questionnaire-9 items, SIAS-6 = Social Interaction Anxiety Scale – items, Mini-SPIN = Mini-Social Phobia Inventory, AUDIT-C = Alcohol Use Disorders Identification Test-Concise, DAST-10 = Drug Abuse Screening Test – 10 items, EQ-5D-5L = EuroQol-5D-5L, PSS-WIS = Presenteeism Scale for Students – Work Impairment Scale, DEQ-SCP = Depressive Experiences Questionnaire - Self-Critical Perfectionism, ECS-R-SF = Revised Experiences in Close Relationships – Short Form, BDI-II = Beck Depression Inventory scale –II, PHQ-4 = Patient Health Questionnaire- 4 items, TiC-P = Treatment Inventory of Costs in Patients, CSQ-8 = Client Satisfaction Questionnaire – 8 items.

For peer review only

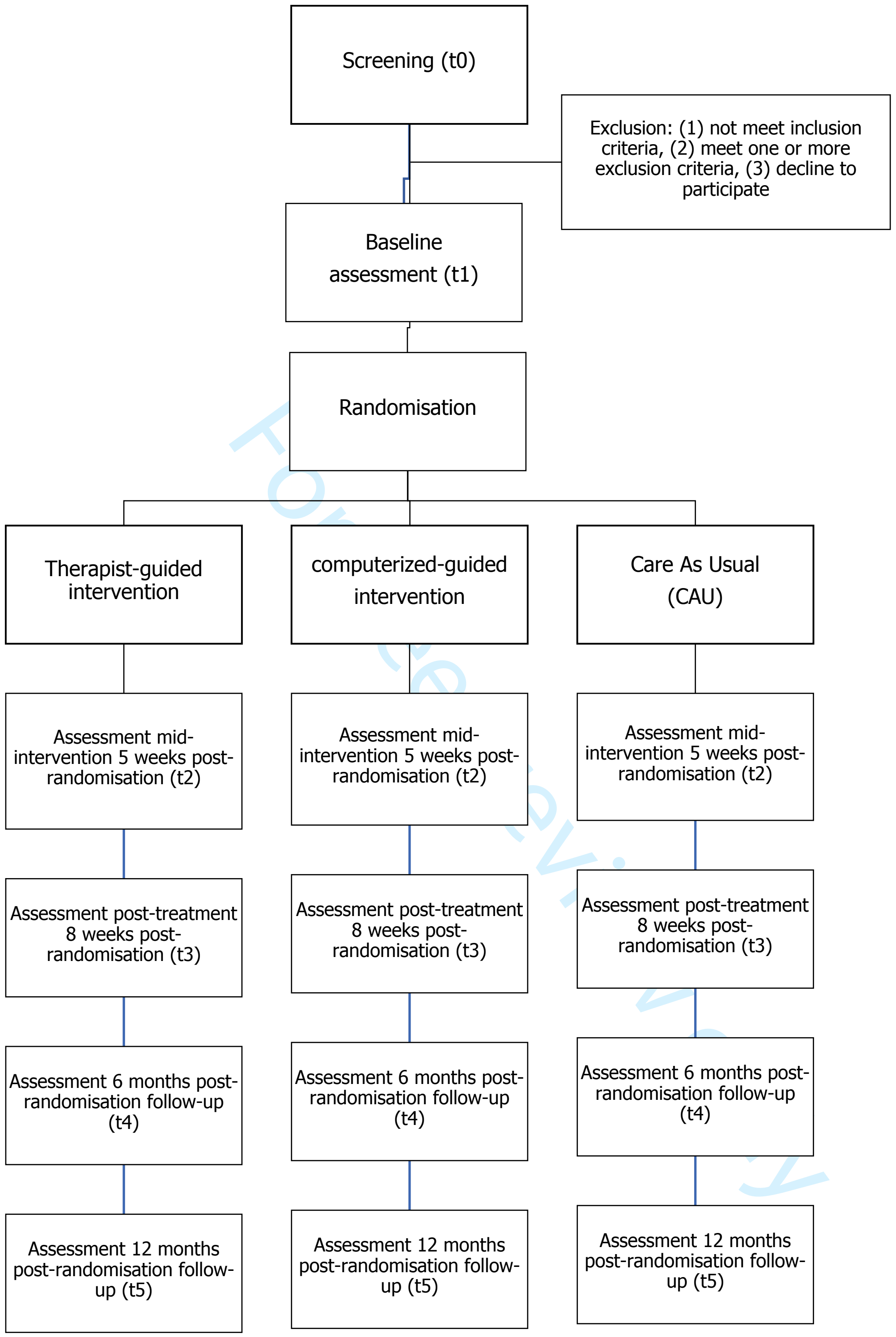

Figure 1. Flow of the study

Reporting checklist for protocol of a clinical trial.

Based on the SPIRIT guidelines.

Instructions to authors

Complete this checklist by entering the page numbers from your manuscript where readers will find each of the items listed below.

Your article may not currently address all the items on the checklist. Please modify your text to include the missing information. If you are certain that an item does not apply, please write "n/a" and provide a short explanation.

Upload your completed checklist as an extra file when you submit to a journal.

In your methods section, say that you used the SPIRITreporting guidelines, and cite them as:

Chan A-W, Tetzlaff JM, Gøtzsche PC, Altman DG, Mann H, Berlin J, Dickersin K, Hróbjartsson A, Schulz KF, Parulekar WR, Krleža-Jerić K, Laupacis A, Moher D. SPIRIT 2013 Explanation and Elaboration: Guidance for protocols of clinical trials. BMJ. 2013;346:e7586

		Reporting Item	Page Number
**Administrative information**			
Title	#1	Descriptive title identifying the study design, population, interventions, and, if applicable, trial acronym	1
1Trial registration	#2a	Trial identifier and registry name. If not yet registered, name of intended registry	2
Trial registration: data set	#2b	All items from the World Health Organization Trial Registration Data Set	1, 2, 8, 10, 11, 12, 18, 19, 20, 30
Protocol version	#3	Date and version identifier	header
Funding	#4	Sources and types of financial, material, and other support	19
Roles and responsibilities: contributorship	#5a	Names, affiliations, and roles of protocol contributors	1, 19

For peer review only

Roles and responsibilities: sponsor contact information	#5b	Name and contact information for the trial sponsor	19
Roles and responsibilities: sponsor and funder	#5c	Role of study sponsor and funders, if any, in study design; collection, management, analysis, and interpretation of data; writing of the report; and the decision to submit the report for publication, including whether they will have ultimate authority over any of these activities	19
Roles and responsibilities: committees	#5d	Composition, roles, and responsibilities of the coordinating centre, steering committee, endpoint adjudication committee, data management team, and other individuals or groups overseeing the trial, if applicable (see Item 21a for data monitoring committee)	18, 19
**Introduction**			
Background and rationale	#6a	Description of research question and justification for undertaking the trial, including summary of relevant studies (published and unpublished) examining benefits and harms for each intervention	5,6,7
Background and rationale: choice of comparators	#6b	Explanation for choice of comparators	5,6,7
Objectives	#7	Specific objectives or hypotheses	7
Trial design	#8	Description of trial design including type of trial (eg, parallel group, crossover, factorial, single group), allocation ratio, and framework (eg, superiority, equivalence, non-inferiority, exploratory)	8
**Methods: Participants, interventions, and outcomes**			
Study setting	#9	Description of study settings (eg, community clinic, academic hospital) and list of countries where data will be	8,9,

For peer review only

		collected. Reference to where list of study sites can be obtained	
Eligibility criteria	#10	Inclusion and exclusion criteria for participants. If applicable, eligibility criteria for study centres and individuals who will perform the interventions (eg, surgeons, psychotherapists)	8
Interventions: description	#11a	Interventions for each group with sufficient detail to allow replication, including how and when they will be administered	10,11,12
Interventions: modifications	#11b	Criteria for discontinuing or modifying allocated interventions for a given trial participant (eg, drug dose change in response to harms, participant request, or improving / worsening disease)	8
Interventions: adherance	#11c	Strategies to improve adherence to intervention protocols, and any procedures for monitoring adherence (eg, drug tablet return; laboratory tests)	9,15,18
Interventions: concomitant care	#11d	Relevant concomitant care and interventions that are permitted or prohibited during the trial	8
Outcomes	#12	Primary, secondary, and other outcomes, including the specific measurement variable (eg, systolic blood pressure), analysis metric (eg, change from baseline, final value, time to event), method of aggregation (eg, median, proportion), and time point for each outcome. Explanation of the clinical relevance of chosen efficacy and harm outcomes is strongly recommended	13, 14, 15, 16, 28, 29
Participant timeline	#13	Time schedule of enrolment, interventions (including any run-ins and washouts), assessments, and visits for participants. A schematic diagram is highly recommended (see Figure)	30
Sample size	#14	Estimated number of participants needed to achieve study objectives and how it was determined, including clinical and statistical assumptions supporting any sample size calculations	16
Recruitment	#15	Strategies for achieving adequate participant enrolment to reach target sample size	18

For peer review only

**Methods: Assignment of interventions (for controlled trials)**			
Allocation: sequence generation	#16a	Method of generating the allocation sequence (eg, computer-generated random numbers), and list of any factors for stratification. To reduce predictability of a random sequence, details of any planned restriction (eg, blocking) should be provided in a separate document that is unavailable to those who enrol participants or assign interventions	9,10
Allocation concealment mechanism	#16b	Mechanism of implementing the allocation sequence (eg, central telephone; sequentially numbered, opaque, sealed envelopes), describing any steps to conceal the sequence until interventions are assigned	8,9,10
Allocation: implementation	#16c	Who will generate the allocation sequence, who will enrol participants, and who will assign participants to interventions	9,10
Blinding (masking)	#17a	Who will be blinded after assignment to interventions (eg, trial participants, care providers, outcome assessors, data analysts), and how	10
Blinding (masking): emergency unblinding	#17b	If blinded, circumstances under which unblinding is permissible, and procedure for revealing a participant's allocated intervention during the trial	n/a
**Methods: Data collection, management, and analysis**			
Data collection plan	#18a	Plans for assessment and collection of outcome, baseline, and other trial data, including any related processes to promote data quality (eg, duplicate measurements, training of assessors) and a description of study instruments (eg, questionnaires, laboratory tests) along with their reliability and validity, if known. Reference to where data collection forms can be found, if not in the protocol	11, 13,14,15,16

For peer review only

Data collection plan: retention	#18b	Plans to promote participant retention and complete follow-up, including list of any outcome data to be collected for participants who discontinue or deviate from intervention protocols	18
Data management	#19	Plans for data entry, coding, security, and storage, including any related processes to promote data quality (eg, double data entry; range checks for data values). Reference to where details of data management procedures can be found, if not in the protocol	19
Statistics: outcomes	#20a	Statistical methods for analysing primary and secondary outcomes. Reference to where other details of the statistical analysis plan can be found, if not in the protocol	16
Statistics: additional analyses	#20b	Methods for any additional analyses (eg, subgroup and adjusted analyses)	16
Statistics: analysis population and missing data	#20c	Definition of analysis population relating to protocol non-adherence (eg, as randomised analysis), and any statistical methods to handle missing data (eg, multiple imputation)	16
**Methods: Monitoring**			
Data monitoring: formal committee	#21a	Composition of data monitoring committee (DMC); summary of its role and reporting structure; statement of whether it is independent from the sponsor and competing interests; and reference to where further details about its charter can be found, if not in the protocol. Alternatively, an explanation of why a DMC is not needed	n/a
Data monitoring: interim analysis	#21b	Description of any interim analyses and stopping guidelines, including who will have access to these interim results and make the final decision to terminate the trial	18,19
Harms	#22	Plans for collecting, assessing, reporting, and managing solicited and spontaneously reported adverse events and other unintended effects of trial interventions or trial conduct	18,19

For peer review only

Auditing	#23	Frequency and procedures for auditing trial conduct, if any, and whether the process will be independent from investigators and the sponsor	n/a
**Ethics and dissemination**			
Research ethics approval	#24	Plans for seeking research ethics committee / institutional review board (REC / IRB) approval	18,19
Protocol amendments	#25	Plans for communicating important protocol modifications (eg, changes to eligibility criteria, outcomes, analyses) to relevant parties (eg, investigators, REC / IRBs, trial participants, trial registries, journals, regulators)	19
Consent or assent	#26a	Who will obtain informed consent or assent from potential trial participants or authorised surrogates, and how (see Item 32)	9
Consent or assent: ancillary studies	#26b	Additional consent provisions for collection and use of participant data and biological specimens in ancillary studies, if applicable	n/a
Confidentiality	#27	How personal information about potential and enrolled participants will be collected, shared, and maintained in order to protect confidentiality before, during, and after the trial	10, 19
Declaration of interests	#28	Financial and other competing interests for principal investigators for the overall trial and each study site	19
Data access	#29	Statement of who will have access to the final trial dataset, and disclosure of contractual agreements that limit such access for investigators	10,19
Ancillary and post trial care	#30	Provisions, if any, for ancillary and post-trial care, and for compensation to those who suffer harm from trial participation	n/a
Dissemination policy: trial results	#31a	Plans for investigators and sponsor to communicate trial results to participants, healthcare professionals, the public, and other relevant groups (eg, via publication, reporting in results databases, or other data sharing arrangements), including any publication restrictions	19

For peer review only

Dissemination policy: authorship	#31b	Authorship eligibility guidelines and any intended use of professional writers	20
Dissemination policy: reproducible research	#31c	Plans, if any, for granting public access to the full protocol, participant-level dataset, and statistical code	n/a
**Appendices**			
Informed consent materials	#32	Model consent form and other related documentation given to participants and authorised surrogates	A
Biological specimens	#33	Plans for collection, laboratory evaluation, and storage of biological specimens for genetic or molecular analysis in the current trial and for future use in ancillary studies, if applicable	n/a

None The SPIRIT checklist is distributed under the terms of the Creative Commons Attribution License CC-BY-ND 3.0. This checklist can be completed online using https://www.goodreports.org/, a tool made by the EQUATOR Network in collaboration with Penelope.ai

For peer review only

Appendix B

Example of automated feedback in the computer-guided condition:

After completing for example session six, all participants receive the same message in the messaging function; "*Dear participant, Congratulations on finishing the sixth session. We know the training is not always easy, so well done. In this session you were once again reminded that practice makes perfect. Keep trying to find the motivation you had when you started and use the tips from all the sessions thus far to work on difficult situations and challenges. You are now starting with the second to last session of the training module. We wish you once again good luck with the next session! Kind regards, the research team*".

Example of feedback in the therapist-guided condition:

An example of feedback on the sixth session; "*Dear [name of participant], Thank you for sending in the exercises from the sixth and second-to-last session. It's great that you have been persistent with this training over the past weeks! You started this session by taking stock of how your challenges from last week went. It is nice to read that you have faced your first challenge. You have taken a first step to actively conquer your fears, well done! Have you thought about rewarding yourself? This week you detected a safety behavior: carrying items to prepare for the worst-case scenarios despite never having to use them. It's great you could identify it, since you can only start making changes once you have become aware of these behaviours! In this session you have chosen to stick with the challenging area you picked last time, which was dealing with situations where there is no clear process like a queue in a busy cafe. It's great that you made a plan to challenge yourself in the coming week! You might benefit from making sure the link is clear between the activities you planned and the challenging area you chose to focus on. The more concrete and specific your plan is, the*

For peer review only

easier it will be to carry out. It is also important to have a detailed plan so you can accurately judge the difficulty of each situation. This way, you can start with easier challenges and build your way up to harder ones. To follow the examples from the previous session, your plan could be something like "Monday: order coffee with a friend (easier), Wednesday: go alone to the doctor's office where there is a clear system for the queue (moderate), Saturday: order coffee on my own (harder)". It is also possible to start with very small tasks: sometimes just imagining the situation can be a useful first step. Don't forget that every attempt is worth being rewarded and deserves respect! Plan something nice or fun for yourself as soon as you have confronted a situation. This week you also completed the optional module on rest and relaxation. I hope these strategies were useful for you and that you can use them going forward. I am also glad to see that you are continuing to plan positive activities to incorporate into your daily life, like going for a walk, getting takeout, or visiting your friends! In the seventh (and for now last) session of this training you will have the opportunity to draw up a plan for the future. You will get to plan exercises for the coming weeks, and we will look back on the training and your successes. I am looking forward to hearing back from you then! Kind regards, [name of coach]".

For peer review only

BMJ Open

Online Computer- or Therapist-guided Cognitive Behavioral Therapy in University Students with Anxiety and/or Depression: Study Protocol of a Randomized Controlled Trial

Journal:	*BMJ Open*
Manuscript ID	bmjopen-2021-049554.R1
Article Type:	Protocol
Date Submitted by the Author:	19-Jul-2021
Complete List of Authors:	Klein, Anke; University of Amsterdam Psychology,<br>Wolters, N. E.; University of Amsterdam, Developmental Psychology<br>Bol, E. J. M.; University of Amsterdam, Developmental Psychology<br>Koelen, J.; University of Amsterdam, Developmental Psychology<br>de Koning, L.; University of Amsterdam, Developmental Psychology<br>Roetink, S. S. M.; University of Amsterdam, Developmental Psychology<br>van Blom, J. J.; University of Amsterdam, Developmental Psychology<br>Pronk, T.; University of Amsterdam Psychology<br>van der Heijde, Claudia; Universiteit van Amsterdam<br>Salemink, Elske; Utrecht University<br>Bolinski, Felix; Vrije Universiteit Amsterdam, Clinical, Neuro- & Developmental Psychology<br>Riper, Heleen; Vrije Universiteit Amsterdam<br>Karyotaki, Eirini ; Vrije Universiteit Amsterdam, Clinical Neuro and Developmental Psychology<br>Cuijpers, Pim; Vrije Universiteit Amsterdam, Department of Clinical Psychology<br>Schneider, S.; Ruhr University Bochum<br>Rapee, Ronald M.; Macquarie University, Psychology<br>Vonk, Peter ; Universiteit van Amsterdam<br>Wiers, Reinout; University of Amsterdam, Developmental Psychology
**Primary Subject Heading**:	Mental health
Secondary Subject Heading:	Addiction
Keywords:	Adult psychiatry < PSYCHIATRY, Anxiety disorders < PSYCHIATRY, Depression & mood disorders < PSYCHIATRY

SCHOLARONE™
Manuscripts

I, the Submitting Author has the right to grant and does grant on behalf of all authors of the Work (as defined in the below author licence), an exclusive licence and/or a non-exclusive licence for contributions from authors who are: i) UK Crown employees; ii) where BMJ has agreed a CC-BY licence shall apply, and/or iii) in accordance with the terms applicable for US Federal Government officers or employees acting as part of their official duties; on a worldwide, perpetual, irrevocable, royalty-free basis to BMJ Publishing Group Ltd ("BMJ") its licensees and where the relevant Journal is co-owned by BMJ to the co-owners of the Journal, to publish the Work in this journal and any other BMJ products and to exploit all rights, as set out in our licence.

The Submitting Author accepts and understands that any supply made under these terms is made by BMJ to the Submitting Author unless you are acting as an employee on behalf of your employer or a postgraduate student of an affiliated institution which is paying any applicable article publishing charge ("APC") for Open Access articles. Where the Submitting Author wishes to make the Work available on an Open Access basis (and intends to pay the relevant APC), the terms of reuse of such Open Access shall be governed by a Creative Commons licence – details of these licences and which Creative Commons licence will apply to this Work are set out in our licence referred to above.

Other than as permitted in any relevant BMJ Author's Self Archiving Policies, I confirm this Work has not been accepted for publication elsewhere, is not being considered for publication elsewhere and does not duplicate material already published. I confirm all authors consent to publication of this Work and authorise the granting of this licence.

For peer review only

Online Computer- or Therapist-guided Cognitive Behavioral Therapy in University Students with Anxiety and/or Depression: Study Protocol of a Randomized Controlled Trial

A. M. Klein[12*], N. E. Wolters[1], E. J. M. Bol[1], J. Koelen[1], L. de Koning[1], S. S. M. Roetink[1], J. J. van Blom[1], T. Pronk[13], C. M. van der Heijde[2], E. Salemink[1], F. Bolinksi[4], H. Riper[4], E. Karyotaki[4], P. Cuijpers[4], S. Schneider[5], R. M. Rapee[6], P. Vonk[2], & R. W. Wiers[1]

1. Addiction Development and Psychopathology (ADAPT)-lab, Developmental Psychology, Department of Psychology, University of Amsterdam
2. Department of Research, Development and Prevention, Student Health Service, University of Amsterdam, Amsterdam, The Netherlands
3. Behavioural Science Lab, Faculty of Social and Behavioural Sciences, University of Amsterdam, Amsterdam, The Netherlands
4. Department of Clinical Neuro- and Developmental Psychology, Amsterdam Public Health Research Institute, Vrije Universiteit Amsterdam, The Netherlands
5. Clinical Child and Adolescent Psychology, Ruhr University Bochum, Germany
6. Centre for Emotional Health, Macquarie University, Australia

* Corresponding authors: A.M. Klein, Ph.D., a.m.klein@uva.nl; Reinout Wiers, Ph.D., r.w.h.j.wiers@uva.nl

For peer review only - http://bmjopen.bmj.com/site/about/guidelines.xhtml

Abstract

Introduction: Emerging adulthood is a phase in life that is associated with an increased risk to develop a variety of mental health disorders including anxiety and depression. However, less than 25% of university students receive professional help for their mental health complaints. Internet-based Cognitive Behavioral Therapy (iCBT) may entail useful interventions in a format that is attractive for university students. The aim of this study protocol is to test the effectiveness of a therapist-guided versus a computer-guided transdiagnostic iCBT program with a main focus on anxiety and depression.

Methods and Analysis: University students with anxiety and/or depressive symptoms will be randomized to a 1) 7-week iCBT program (excluding booster-session) with therapist feedback, 2) the identical iCBT program with computer feedback only or, 3) care as usual. Participants in the care as usual condition are informed and referred to conventional care services and encouraged to seek the help they need. Primary outcome variables are self-reported levels of anxiety as measured with the General Anxiety Disorder-7 (GAD-7), and self-reported levels of depression as measured with the Patient Health Questionnaire-9 (PHQ-9). Secondary outcomes include treatment adherence, client satisfaction, medical service use, substance use, quality of life and academic achievement. Assessments will take place at baseline (t1), mid-treatment (t2), post-treatment (t3), at 6 months (t4) and 12 months (t5) post-baseline. Social anxiety and perfectionism are included as potentially important predictors of treatment outcome. Power calculations are based on a 3 (group) x 3 (measurement: pre-, mid- and post treatment) interaction resulting in an aimed sample of 276 participants. Data will be analyzed based on intention-to-treat, and per protocol samples using mixed linear models.

For peer review only - http://bmjopen.bmj.com/site/about/guidelines.xhtml

Ethics and Dissemination: The current study was approved by the METC of the Academic Medical Center, Amsterdam, The Netherlands (number: NL64929.018.18). Results of this trial will be published in peer-reviewed journals.

Trial registration: Dutch Trial register number: NL7328.

(307 words)

Keywords: Anxiety, Depression, eHealth, online Cognitive Behavioral Treatment (iCBT), Therapist-guided versus computer-guided, Randomized controlled trial, University students

For peer review only

For peer review only - http://bmjopen.bmj.com/site/about/guidelines.xhtml

Article Summary

Strength and limitations of this study

- The current study protocol describes a Randomized Controlled Trial (RCT) focused on university students with anxiety and/or depressive symptoms.
- A transdiagnostic iCBT program, with either therapist guidance or computerized guidance, is compared with care as usual (3 arms RCT)
- The procedure of the trial is developed to make it easily accessible and anonymous, and the full procedure is online.
- The therapists are not fully blinded to the treatment condition.
- The post-treatment outcomes rely on self-reports of the participants.

For peer review only

Background

Emerging adulthood is a crucial stage in life that includes the transition from the teenage years to full-fledged adulthood (18-25 years). It is a phase during which many young adults attend university to receive education and training to obtain a degree that will be the basis for their adult working life [1]. It is also the phase that is associated with an increased risk of developing a variety of mental health disorders, such as anxiety and depression [2;3;4] whereby comorbidity is the rule rather than the exception [5]. Recent studies found that between 16-46% of all students suffer from mental health disorders [2;6]. Moreover, these problems have been associated with poorer academic performance, academic dropout, decreased labor market functioning, and poorer social and health outcomes [6;7;8;9;10]. Early detection and effective intervention are thus crucial with regard to the long-term prognosis of mental disorders, especially as untreated mental disorders have a mean duration between 4 and 23 years [11].

Online treatment programs may be useful as an intervention in a format that is particularly attractive for university students, given the fact that less than 25% of students with mental health problems utilize available face to face services [12]. University students might avoid help due to several reasons including fear of stigmatization, lack of perceived urgency, preference to deal with it themselves, threat to identity formation and goal setting, and/or skepticism regarding treatment effectiveness [13;14]. This group may be more likely to seek help if an effective, more flexible, easily accessible, and anonymous intervention strategy is provided. A scoping review by [15] concluded that internet therapies have (untapped) potential to provide better information about mental health and may improve the cost efficacy of mental health services. Moreover, their study indicated that young people, in general, prefer eHealth treatments relative to face-to-face services.

For peer review only - http://bmjopen.bmj.com/site/about/guidelines.xhtml

Several studies demonstrated that the effectiveness of guided internet-based Cognitive Behavioral Therapy (iCBT) programs for anxiety and depression are comparable to conventional (face-to-face) CBT [for meta-analyses 16; 17]. There is also some evidence for the effectiveness of internet interventions for young adults and university student populations with various mental health problems, including depression, anxiety, eating disorders, stress, alcohol, and sleep problems [e.g.,18; 19; 20; 21; 22]. For example, a recent meta-analysis [20] focused on university students with mental problems who self-selected a psychological online intervention (guided / unguided). They found small intervention effects for depression (g = 0.18, 95% confidence interval [CL; 0.08, 0.27]) and anxiety (g = 0.27, 95% confidence interval [CL; 0.13, 0.40]) in university students. It should be noted, however, that the effects on anxiety were not significant anymore after adjusting for publication bias. Harrer and colleagues [20] concluded that clearly more research is needed to explore ways to increase treatment effectiveness for university students and to study predictors of treatment outcome.

The current study includes a group of university students and focuses on two of the most common mental disorders in this group, namely anxiety and depression [2]. As comorbidity is high, we will use a transdiagnostic iCBT intervention which has the advantage to target both disorders at the same time and thereby might increase treatment effectiveness [for a systemic review and meta-analysis see 23]. Moreover, iCBT programs can be provided with or without additional personal guidance. Guidance usually implies written or verbal feedback by the therapist to the patient's homework assignments, and other forms of (safe) communication (e.g., chat, telephone) within an online environment with varying degrees of interactive features [24]. Both approaches (with and without personal guidance) have shown to be efficacious, although programs with (therapist) guidance generally report a higher effect size [for meta-analyses, 17; 25], even though it should be noted that a recent meta-analysis [20] found no difference between personal or no personal guidance. To date, little is known

For peer review only - http://bmjopen.bmj.com/site/about/guidelines.xhtml

about the effectiveness for therapist-guided versus a computer-guided version of the same intervention tested *within* the same study when applied to a university student sample with mental health concerns [see also 20; 26; 27]. This could also have important health economic implications considering the additional cost of human support. Therefore, the current study tests this transdiagnostic iCBT for anxiety and depression in three groups; a therapist-guided online self-help version, a computer-guided version of the same program and care as usual. Primary outcomes were anxiety and depression; secondary measures were included to evaluate treatment adherence and satisfaction, academic achievement and medical service use.

Internet-based programs, either therapist-guided with personal feedback or computer-guided with automated feedback, might suit some individuals better than others. It is important to focus on possible treatment predictors (e.g., including baseline symptoms severity, gender, personality factors and comorbidity), as this may help us understand more about the heterogeneity of treatment response [e.g., 20; 28]. Specially, a recent meta-analysis [20] found limited effectiveness on anxiety in university students in their meta-analysis. The current study will focus on the possible role of social anxiety and perfectionism, as there is some evidence that adults and children with social anxiety might benefit less from general CBT programs [for meta-analyses see 29; 30]. Likewise, self-critical perfectionism is a transdiagnostic personality factor that is generally associated with worse treatment outcome, including CBT [31]. Importantly, individuals with self-critical perfectionism have poorer social relationships, which in turn negatively impacts therapeutic progress [32].

The aim of this study is to test the effectiveness of a therapist-guided versus a computer-guided transdiagnostic iCBT program with a focus on anxiety and depression. Based on previous studies in the general population [17], we hypothesize that the therapist-guided iCBT and computer-guided iCBT conditions are both significantly more effective

For peer review only - http://bmjopen.bmj.com/site/about/guidelines.xhtml

than care as usual. In addition, we expect that the therapist-guided iCBT condition is significantly more effective than the computer-guided iCBT condition; an effect we expect to be mediated by higher treatment adherence.

Methods

Study design

The current study includes a Randomized Controlled Trial (RCT) design with three arms, consisting of (1) a therapist-guided transdiagnostic iCBT program with personalized feedback, (2) a computer-guided version of the same CBT program with automated feedback, and (3) care as usual (CAU). We used the SPIRIT reporting guidelines for this study [33; see Appendix A].

Eligibility criteria

All student scoring 16 or higher on the Center for Epidemiological Studies Depression Scale (CES-D) [34] and/or a score of 5 or higher on the General Anxiety Disorder-7 (GAD-7) [35] were included in the study. There is no higher end cut-off. Participants are excluded from the study if they 1) are younger than 16 years of age, 2) currently receive psychological treatment (as described by the APA dictionary) for anxiety and/or depression, 3) do not have access to a stable internet connection for the next two months, 4) if they meet the diagnostic criteria for (recent or current) psychotic disorder according to the Mini International Neuropsychiatric Interview (M.I.N.I) [36], 5) if they are at active high risk for suicide based on the MINI, and 6) if they do not give informed consent. Participants meeting exclusion criteria 4 and/or 5 are referred to local health care facilities.

Recruitment

For peer review only - http://bmjopen.bmj.com/site/about/guidelines.xhtml

All participants were recruited at the University of Amsterdam (UvA), The Netherlands. The UvA is a public research university located in Amsterdam. It is one of the largest research universities in Europe with 31186 students enrolled in 2018 and includes seven faculties: Humanities, Social and Behavioural Sciences, Economics and Business, Science, Law, Medicine, and Dentistry. Recruitment for the study is done in two phases: a first broad screening phase for all university students studying at the UvA and a second more specific screening of eligible students who showed interest to participate. In the first broad screening phase, all students enrolled at the UvA receive an invitation via email that are sent centrally from the study platform. In addition, study advisors and study counselors of the UvA are informed about the study and are asked to refer students who are interested in participating in the study to the research team so that they can be invited by email.

This first broad screening invitation email contains general information about the study and a unique link to fill out the screening questionnaire. This screening questionnaire takes approximately 20 minutes to complete and also includes other questionnaires related to the mental health of university students. This screening questionnaire was developed in collaboration with other universities to collect multicenter data. Participants who click on the invitation link are referred to the project platform. Here, they find an information letter and are asked to give online informed consent to the research team. This information letter explicitly states that participation is voluntary and that participants can withdraw at any time without consequences. Next, participants are asked to create an account after which they are directed to the screening questionnaire. Following the original invitation, two reminder emails are sent one- and two weeks after the first invitation. As the study runs for three academic years (2019-2021), participants are asked at the beginning of the screening if they consent to being invited again later. Moreover, participants can also indicate that they do not want to receive any emails again.

For peer review only - http://bmjopen.bmj.com/site/about/guidelines.xhtml

In the second more specific screening phase, university students who score above the cut-off on either anxiety (GAD-7) and/or depressive symptoms (CES-D) receive an email with an online information brochure and informed consent document [see also: 34; 35; see Appendix B]. After informed consent is given by the participant, they are called by a trained clinical research assistant to check all of the inclusion and exclusion criteria. A telephonic diagnostic interview [M.I.N.I.; 36] is administered to establish possible diagnoses with respect to mood and anxiety disorders, bipolar disorder, psychosis, and suicidal ideation (not fulfilling the criteria for an anxiety and/or mood disorder is not an exclusion criterium).

Randomization, blinding and treatment allocation

Participants are randomly assigned to either the therapist-guided online intervention, the computer-guided online intervention or care as usual (control) condition (1:1:1 allocation ratio) directly following the baseline measurement. Randomization is stratified by gender and anxiety and /or depressive symptoms (based on the cut-off scores on the screening questionnaire). Sequence generation is based on computer-generated random numbers and are allocated by an automatic system.

Due to the nature of the intervention, participants and researchers cannot be completely blinded to the assigned treatment condition, though note that the measurements in the intervention are blinded. Researchers are aware of the participants ’condition because they provide participants with feedback in the therapist-guided condition. In the computer-guided condition feedback is automatically generated by the system, but therapists do send reminders to this group as part of the intervention.

Intervention

For peer review only

The online transdiagnostic intervention that is used in this study, 'ICare Prevent', was originally developed by [37] for the general German-speaking population, and translated and adapted by [38] and [39] for a Dutch undergraduate student population into Dutch and English. The intervention was based on other effective protocols (e.g. [40, 41, 42, 43]) and can be seen as a variant of an iCBT protocol for which several studies have proven its effectiveness. The intervention is provided to the participants through the eHealth platform Minddistrict. This platform enables researchers and healthcare providers to provide digital therapy to their participants and patients. On this platform, e-coaches create a personal account for each participant directly following the baseline assessment and randomization. Once the personal account has been created, the participant receives an email with information how to activate the account. Once the account is activated, the participant can start with the intervention. The platform monitors the progress of the participants, e-coaches provide support for the participants in the therapist-guided treatment condition, and respond to questions from the participants with the messaging function. Data processing and storage are in accordance with the ISO 27000 and NEN 7510 norms. A data processing agreement that complies with the GDPR has been signed between eHealth provider Minddistrict and the University of Amsterdam.

The intervention is based on basic cognitive behavioral therapy principles for anxiety and depression with components of psychoeducation, including online exercises and home assignments. The intervention consists of 7 regular sessions (45 - 60 minutes per session) and 1 booster session (4 weeks after the completion of the last session). From the second session onward, participants are able to follow eight additional optional modules based on their personal needs, including sleep, perfectionism, alcohol use, reduce rumination, self-worth, acceptance, appreciations, and rest and relaxation. They can decide to choose one additional module per session, and they are free to repeat this module or choose other modules in later

For peer review only - http://bmjopen.bmj.com/site/about/guidelines.xhtml

sessions. In session five and six, users decide to follow either content directed at changing negative cognitions, or exposure to feared situations. Participants are free to decide when they would like to start a treatment session and if they want to do an additional module, but it is advised to do at least one and no more than two treatment sessions per week. For a full description of the intervention, see [39].

Therapist guided intervention versus computer-guided intervention

During the intervention, participants in both conditions receive online support in the form of messages or notifications in the messaging function (e-mails). In both conditions, participants receive up to three weekly reminders via the messaging function when they are inactive. Moreover, participants in both conditions can use the messaging function to ask questions. All participants can ask technical or usability related questions. There is one key difference between the two conditions: Participants in the computer-guided iCBT condition receive automatically generated feedback messages (see Appendix C for an example). In contrast, in the therapist-guided condition, the e-coaches (trained psychologists on minimal BA level) provide detailed feedback based on participants' output of the sessions, and this feedback is displayed at the bottom of the session they completed (see Appendix C for an example). The coach spends approximately 30 minutes on providing feedback per session and intends to give feedback within 5 working days after the session is completed by the participant. During the trial, training and weekly supervision for the e-coaches who are guiding the participants is provided by a licensed mental health care psychologist.

Care as usual

Participants in the care as usual condition are informed and referred to conventional care services and encouraged to seek the help they need. Medical services used by the participants during the RCT are monitored through self-report questions at t3, t4 and t5.

For peer review only - http://bmjopen.bmj.com/site/about/guidelines.xhtml

Participants in the care as usual condition are assessed at the same time points as the two intervention conditions including the weekly questionnaires during the first 7 weeks.

Suicide risk monitoring

All participants receive an information brochure with all relevant contact information as part of the information package they receive prior to the study. In addition, the brief version of the PHQ-9, the PHQ-4 [44] is administered before each treatment session, or weekly via email (care as usual). Suicide risk is monitored with the help of a suicide risk protocol that was created specifically for this study [Klein et al., 2018. *Suicide protocol*]. Suicide risk is monitored using item 3 ('feeling down, depressed, or hopeless') of the PHQ-4 and item 9 ('thought that you would be better off dead, or of hurting yourself') of the Beck Depression Inventory (BDI)-II scale [45]. If deemed necessary, participants are called and referred to appropriate services, including their GP or 113zelfmoordpreventie (online platform for suicide prevention). In addition, the participant also receives a pop-up message on the questionnaire page with relevant contact information right after filling in the questionnaire (i.e., where they can find help if needed with relevant contact information).

Assessments

The RCT includes five assessment points: (1) a baseline assessment including a diagnostic interview and questionnaires, (2) a mid-treatment questionnaire, 5 weeks after baseline, (3) a post-treatment questionnaire, 8 weeks after baseline and (4) follow-up measurements, 6 months, and (5) 12 months after baseline (see also Figure 1). In addition, prior to each treatment session, or weekly in the CAU-condition, participants fill out a short online questionnaire. This questionnaire briefly assesses symptom severity and monitors suicidal ideation.

For peer review only - http://bmjopen.bmj.com/site/about/guidelines.xhtml

Primary outcomes

The Generalized Anxiety disorder-7 (GAD-7) [35] and the Patient Health Questionnaire-9 (PHQ-9) [46] are the primary outcomes in the study and assess respectively *anxiety and depressive symptoms* on a continuous scale. The 7-item GAD is a self-report questionnaire measuring anxiety symptoms. All items are scored on a scale from 0 (not at all') to 3 ('nearly every day'), with total scores ranging from 0 to 21. A higher score on the questionnaire is associated with a more severe experience of anxiety symptoms. The GAD-7 questionnaire has good psychometric properties including a good test re-test reliability (ICC = .83) [35] and a good internal consistency (.79 < α < 0.91) [47]. The PHQ-9 questionnaire is used to screen for self-reported symptoms of depression. The 9 items are scored on a 0-3 scale, resulting in a total score between 0 and 27. A higher score on the scale indicates a more severe experience of depressive symptoms. The PHQ-9 is a valid and reliable tool with good sensitivity (.88), and high specificity (.94) [48; 49] (see Table 1 for an overview of all questionnaires on all time points).

Secondary outcomes

Alcohol and *drug use* are measured with respectively the Alcohol Use Disorders Identification Test-Concise (AUDIT-C) [50; 51] and the Drug Abuse Screening Test – 10 items (DAST-10) [52]. The AUDIT-C is a brief screening tool for identifying early and/or heavy alcohol use. The scale consists of 3 items, all scored on a 0-4 scale, resulting in a total score ranging from 0-12. The DAST-10 consists of 10 items. Item responses are 'yes' or 'no'. Higher scores indicate a more severe level of drug abuse problems. The DAST-10 reports high validity (α = .94) and reliability in a broad range of settings and populations [53].

For peer review only - http://bmjopen.bmj.com/site/about/guidelines.xhtml

Quality of life is measured with the EuroQol-5D-5L scale (EQ-5D-5L) [54]. The EuroQol-5D-5L scale assesses health-related wellbeing on five dimensions: mobility, self-care, ordinary activities, discomfort, and mood state. The sixth item of the EQ-5D-5L is a VAS scale, ranging from 0-100, asking the participant to rate their health perception at that moment. The EQ-5D-5L has high levels of acceptability and sensitivity [55; 56].

Academic achievement is measured with objective and subjective measures. Students are asked to report their number of European Credit Transfer System (ECTS) as an objective measure of their academic performance. Additionally, the work impairment subscale of the Presenteeism Scale for Students (PSS-WIS) [57] is administered to assess subjective academic achievement on a 5-point Likert scale ranging from 'always' (0) to 'never' (5). The PSS-WIS is a valid (ICC=.88 [95% CI, $p < .001$]) and reliable (test-re-test reliability $r = .80$, $p < .001$) self-report measure to screen for impaired work performance due to (mental) health problems [57].

Medical service use is measured with two items from the Treatment Inventory of Costs in Patients with mental disorders (TiC-P) [58]. The first item includes the frequency of contact with conventional care services (e.g., general practitioner, study advisor, psychologist, medical specialist). The second item includes the use of medication during the length of the treatment period.

Client satisfaction with treatment is measured with the Client Satisfaction Questionnaire – 8 items (CSQ-8) [59; 60]. The CSQ-8 consists of 8 items scored on a 1 ('quite dissatisfied')-4 ('very satisfied') scale, with total scores ranging between 8-32. The CSQ-8 is a standardized satisfaction measure reporting very good internal consistency (α=.83-.93) and high validity [61].

Finally, following previous studies [for a systematic review, 62] *treatment adherence* is measured by tracking the activities in Minddistrict. We collect the total number of modules

For peer review only - http://bmjopen.bmj.com/site/about/guidelines.xhtml

completed, time spent per module and the number of times the participants log into the Minddistrict platform.

Predictors of treatment outcome

Social anxiety is measured with the Social Interaction Anxiety Scale-6 item (SIAS-6) [63] and the brief Mini-Social Phobia Inventory (Mini-SPIN) [64]. The brief Mini-Social Phobia Inventory (Mini-SPIN) [64] is included to screen for generalized social anxiety disorder. The scale shows strong sensitivity (93.8%) and good internal consistency (α = 0.85) [65; 66]. Besides the administration on t1-t5, the Mini-SPIN is administered before each treatment session, or weekly via email (care as usual), to monitor the social anxiety status of the participant.

Perfectionism is measured with the short version of the Self-Critical Perfectionism subscale of the Depressive Experiences Questionnaire (DEQ-SCP) [67]. The subscale consists of 12 items. The DEQ-SCP scale is a simple, valid scale demonstrating good psychometric properties, in both clinical and student samples [68].

Besides the inclusion of social anxiety and perfectionism, we include several variables to study possible predictors of treatment outcome including demographic variables (i.e. age, gender, study direction, faculty, national/international student), baseline severity, personality factors, number of sessions completed/adherence rate and comorbidity for explorative analyses.

Statistical analyses

Sample size calculation. The power analysis is based on the mixed factors 3x3 interaction difference between the three treatment arms and three time points (pre- mid- and post measurement). We anticipate a small interaction effect (Cohen's f = .1) at post-treatment

For peer review only - http://bmjopen.bmj.com/site/about/guidelines.xhtml

based on a recent meta-analysis that focused on the effectiveness of therapist-guided versus computer-guided psychotherapy in treating depression and anxiety [17]. We determined a statistical power (1-ß) of .8 and corrected alpha of α = .05. With a two-tailed hypothesis, we need 204 participants per intervention group to be able to detect a statistically significant result. Previous literature has shown that web-based interventions have a relatively high reported range of drop-out in university students, varying between approximately 30% - 40% post treatment [e.g. 20] Calculating with a dropout rate of 35%, the minimum required total sample for the RCT is N_{total} = 276 participants (with a ratio of 1:1:1), with 92 participants in each group.

Clinical analyses. Mixed Linear Models analyses will be conducted with SPSS and R. To get a more realistic picture of the true efficacy of treatment and completer analyses will also be performed for those participants who adhere to the protocol.

Predictor analyses. Predictors of treatment outcome will be investigated on an exploratory basis with pre-defined predictors: social anxiety and perfectionism, baseline symptoms severity, gender, personality factors and comorbidity). Analyses will be conducted using regression analyses, with interaction effects and structural equation modelling.

Patient and public involvement

A focus group of university students helped with the design of the study. This group also helped with writing the consent letters, the website texts and checked the clearness of all instructions on the online platform. All university students of the UvA will receive an email with the (anonymized) results at the group level when the project is finished.

Discussion

For peer review only - http://bmjopen.bmj.com/site/about/guidelines.xhtml

Even though many university students face mental problems, only relatively few students utilize available services [12]. Internet Cognitive Behavioral Therapy (iCBT) programs are easily accessible, flexible, and relatively anonymous [24]. Hence, they might be a good alternative for reaching out to this group [e.g., 14]. The current study aims to compare the effectiveness of online interventions for elevated levels of anxiety and/or depression in university students enrolled at the University of Amsterdam: a therapist-guided transdiagnostic iCBT program, a computer-guided version of the same program, and care as usual. Several moderators are included that may potentially amplify or attenuate treatment response, and so may provide new knowledge on effectiveness for different subgroups.

Strengths of this study are the use of a transdiagnostic iCBT program, its large sample size, and the generalizability of the findings since exclusion criteria are minimal (e.g., there are almost no exclusion criteria concerning co-morbid disorders; we created a Dutch and English version so that both national and international student can participate). Moreover, the current iCBT program is flexible, because participants can complete sessions at their own pace and it includes separate modules focusing on related problems (e.g., stress, sleep, alcohol use). This flexibility has both strengths and limitations. On the one hand, participants will not feel pressured, they can work on a session whenever they have time do to so, and they have freedom in choosing additional modules that fit their situation. On the other hand, this might result in a different therapeutic process for each participant, which makes it more difficult to compare the different groups. Also, this flexibility makes it more difficult to investigate the effective components of the program. One way to investigate this is to follow the course of the symptoms by analyzing the pre-session questionnaire. Some participants might find it difficult to stay motivated or decide between the different treatment module options. Therefore, one of the limitations of this study is a potentially high degree of drop-out, especially in the computer-guided program and in the care as usual condition [25]. To

For peer review only - http://bmjopen.bmj.com/site/about/guidelines.xhtml

minimize drop-out, participants receive up to three weekly reminders via the messaging function when they are inactive. In addition, we provide all participants with technical support and suicide risk monitoring. Also, there may be a potential confound of "feedback length", as the feedback in the therapist-guided condition is textually longer than the automated feedback in the computer-guided condition. Due to the large sample size but limited human resources, and our priority to maintain the privacy of participants, all measurements are self-reported, and may therefore be more subjective than clinician-rated reports. Moreover, participants may not report certain information, for example whether they receive face-to-face treatment or counseling for their complaints during the current study. Finally, our automated study design allows for blinded assessments throughout the intervention, which on the downside may lead to some assessments not being aligned with the intervention phase (e.g., "mid-treatment" questionnaires being completed before session 4 has been reached.)

In sum, the current trial is unique as it compares two versions of the same transdiagnostic iCBT intervention; a therapist-guided versus a computer-guided version in a large sample that includes a one-year follow-up. This study will therefore improve our understanding of the need of individualized feedback for computer-based-interventions. The findings will significantly add to the knowledge on the treatment of depression and anxiety in university students and will give insight into treatment predictors/moderators that may determine why some students benefit and others do not.

Declarations

Ethical approval, consent to participate and dissemination

The current study was approved by the METC of the Academic Medical Center, Amsterdam, The Netherlands (number: NL64929.018.18), and follows all Dutch ethical legislations. Any

For peer review only

unintended effects will be reported to the METC committee. All procedures are in accordance with the latest version of the Helsinki declaration, and comply to the GDPR rules (2018). All protocol changes will be reported to the ethical committees, and trial participants per email. Active informed consent from participants is asked for the entire procedure. Results of this trial will be published in peer-reviewed journals.

Data protection, data access and data management

The current project fulfils all GDPR requirements, data was recruited in The Netherlands. All authors have access to the data. A data management plan was developed and approved by an external data privacy officer of the University of Amsterdam. Data and the data-management plan will be shared upon request by contacting the corresponding author.

Competing interests

The authors state that they have no conflict of interest.

Funding

This work was supported by a grant from the University of Amsterdam (Spui 21, 1012GC, +31205251400) awarded to Dr. A. M. Klein (Co-PI), Drs. Peter Vonk (PI; p.vonk@uva.nl; Oude Turfmarkt 151, 1012LA, Amsterdam, University of Amsterdam), Claudia van der Heijde (Co-PI) and Prof. Reinout Wiers (PI; r.w.h.j.wiers@uva.nl; Nieuwe achtergracht 129, 1001NK, Amsterdam, University of Amsterdam). Award/grant number non applicable. A steering group from the University of Amsterdam monitors the processes of the entire trial and approves mid-term evaluation.

Author Contributions

For peer review only - http://bmjopen.bmj.com/site/about/guidelines.xhtml

RW (PI), PV (PI), AK (Co-PI) and CvdH (Co-PI) received funding for the project. AK, RW, PV, CvdH, NW, EB, JK, SR, JvB, TP, ES, FB, HR, EK, PC, SN and RP designed of the study. AK drafted the main part of the manuscript, NW and LdK assisted in writing parts of the manuscript under supervision of AK. EB, JL, LdK, SM, JvB, CvdH, ES, FB, HR, EK, PC, SS, RR, PV and RW provided critical feedback for important intellectual content on earlier versions of the manuscript. AK, SR, NW, JK, EB and JvB coordinated the data recruitment and data collection. All authors approved the final version of the manuscript for submission and all authors agreed to be accountable for all aspects of the work.

Acknowledgements

We would like to thank the student psychologists, the student deans, colleagues of academic affairs, the colleagues from the Behavioural Science Lab (BSL) the student advisory boards who were involved in the focus group from the University of Amsterdam and M. van der Hoff and R. Atteveld for their help with the design of the project.

Trial registration and Trial Status

Dutch Trial register number: NL7328. Registered 10 October 2018, https://www.trialregister.nl/trial/7328. The current protocol is the first version. The starting date of the recruitment is 31st January 2019 and runs until March 2022. Dr. A. M. Klein is the contact person for both public and scientific queries: uvacare@uva.nl, +205252878, Oude Turfmarkt 151, 1012LA Amsterdam.

References

For peer review only - http://bmjopen.bmj.com/site/about/guidelines.xhtml

1 Arnett JJ. Emerging adulthood: A theory of development from the late teens through the twenties. *Am Psychol*2000;55(5):469-480.

2 Auerbach RP, Mortier P, Bruffaerts R, et al. WHO World Mental Health Surveys International College Student Project: Prevalence and distribution of mental disorders. *Journal of abnormal psychology*2018;127(7):623-638.

3 De Girolamo G, Dagani J, Purcell R, et al. Age of onset of mental disorders and use ofmental health services: needs, opportunities and obstacles. *Epidemiol Psychiatr Sci*2012;1;21(1):47-57.

4 Zivin K, Eisenberg D, Gollust SE, et al. Persistence of mental health problems and needs in a college student population. *J Affect Disord*2009;117(3):180-5.

5 Auerbach RP, Mortier P, Bruffaerts R, Alonso J, Benjet C, Cuijpers P, Demyttenaere K, Ebert DD, Green JG, Hasking P, Lee S. Mental disorder comorbidity and suicidal thoughts and behaviors in the World Health Organization world mental health surveys international college student initiative. *Int J Methods Psychiatr Res*2019;28(2):e1752.

6 Auerbach RP, Alonso J, Axinn WG, et al. Mental disorders among college students in the World Health Organization world mental health surveys. *Psychol Med*2016;46(14):2955-70.

7 Alonso J, Mortier P, Auerbach RP, et al Severe role impairment associated with mental disorders: results of the WHO world mental health surveys international college student project. *Depress Anxiety*2018;35(9):802-14.

8 Bolinski F, Boumparis N, Kleiboer A, et al. The effect of e-mental health interventions on academic performance in university and college students: A meta-analysis of randomized controlled trials. *Internet Interv*2020:100321.

9 McGorry PD, Purcell R, Goldstone S, et al. Age of onset and timing of treatment for mental and substance use disorders: implications for preventive intervention strategies and models of care. *Curr Opin Psychiatry*2011;24(4):301-6.

For peer review only - http://bmjopen.bmj.com/site/about/guidelines.xhtml

10 Kessler RC, Berglund PA, Bruce ML, et al. The prevalence and correlates of untreated serious mental illness. *Health Serv Res*2001;36(6 Pt 1):987-1007.

11 Wang PS, Berglund P, Olfson M, et al. Failure and delay in initial treatment contact after first onset of mental disorders in the National Comorbidity Survey Replication. *Arch Gen Psychiatry*2005;62(6):603-13.

12 Buchanan JL. Prevention of depression in the college student population: a review of the literature. *Arch Psychiatr Nurs*2012;26(1):21-42.

13 Eisenberg D, Hunt J, Speer N, et al. Mental health service utilization among college students in the United States. *J Nerv Ment Dis*2011;199(5):301-8.

14 Van der Heijde CM, Vonk P, Meijman FJ. Self-regulation for the promotion of student health. Traffic lights: the development of a tailored web-based instrument providing immediate personalized feedback. *Health Psychol Behav Med*2015;3(1):169-89.

15 Boydell KM, Hodgins M, Pignatiello A, et al. Using technology to deliver mental health services to children and youth: a scoping review. *J Can Acad Child Adolesc Psychiatry*2014;23(2):87-99.

16 Carlbring P, Andersson G, Cuijpers P, et al. Internet-based vs. face-to-face cognitive behavior therapy for psychiatric and somatic disorders: an updated systematic review and meta-analysis. *Cogn Behav Ther*2018;47(1):1-8.

17 Cuijpers P, Noma H, Karyotaki E, et al. Individual, group, telephone, self-help and internet-based cognitive behavior therapy for adult depression: A network meta-analysis of delivery methods. *JAMA Psychiatry*2019;76:700-7.

18 Davies EB, Morriss R, Glazebrook C. Computer-delivered and web-based interventions to improve depression, anxiety, and psychological well-being of university students: a systematic review and meta-analysis. *J Med Internet Res*2014;16(5):e130.

For peer review only

19 Ebert DD, Zarski AC, Christensen H, et al. Internet and computer-based cognitive behavioral therapy for anxiety and depression in youth: a meta-analysis of randomized controlled outcome trials. *PloS One*2015;10(3):e0119895.

20 Harrer M, Adam SH, Baumeister H, et al. Internet interventions for mental health in university students: A systematic review and meta-analysis. *Int J Methods Psychiatr Res*2019;28(2):e1759.

21 Sethi S, Campbell AJ, Ellis LA. The use of computerized self-help packages to treat adolescent depression and anxiety. *J Technol Hum Serv*2010;28(3):144-60.

22 Riper H, van Straten A, Keuken M, et al. Curbing problem drinking with personalized-feedback interventions: a meta-analysis. *Am J Prev Med*2009;36(3):247-55.

23 Newby JM, McKinnon A, Kuyken W, et al. Systematic review and meta-analysis of transdiagnostic psychological treatments for anxiety and depressive disorders in adulthood. *Clin Psychol Rev*2015;40:91-110.

24 Andersson G, Cuijpers P, Carlbring P, et al. Guided Internet-based vs. face-to-face cognitive behavior therapy for psychiatric and somatic disorders: a systematic review and meta-analysis. *World Psychiatry*2014;13(3):288-95.

25 Richards D, Richardson T. Computer-based psychological treatments for depression: a systematic review and meta-analysis. *Clin Psychol Rev*2012;32(4):329-42.

26 Berger T, Hämmerli K, Gubser N, et al. Internet-based treatment of depression: a randomized controlled trial comparing guided with unguided self-help. *Cog Behav Ther*2011;40(4):251-66.

27 Renfrew ME, Morton DP, Morton JK, et al. The Influence of Three Modes of Human Support on Attrition and Adherence to a Web-and Mobile App–Based Mental Health Promotion Intervention in a Nonclinical Cohort: Randomized Comparative Study. *J Med Internet Res*2020;22(9):e19945.

For peer review only - http://bmjopen.bmj.com/site/about/guidelines.xhtml

28 Kessler RC, Van Loo HM, Wardenaar KJ, et al. Using patient self-reports to study heterogeneity of treatment effects in major depressive disorder. *Epidemiol Psychiatr Sci*2017;26(1):22-36.

29 Hudson JL, Rapee RM, Lyneham HJ et al. Comparing outcomes for children with different anxiety disorders following cognitive behavioural therapy. Behav Res Ther2015;72:30-37.

30 Norton PJ, & Price, EC. A meta-analytic review of adult cognitive-behavioral treatment outcome across the anxiety disorders. J Nerv Ment Dis2007;195(6):521-31.

31 Löw CA, Schauenburg H, Dinger U. Self-criticism and psychotherapy outcome: A systematic review and meta-analysis. *Clin Psychol Rev*2020;75:101808.

32 Shahar G, Blatt SJ, Zuroff DC, et al. Perfectionism impedes social relations and response to brief treatment for depression. *J Soc Clin Psychol*2004;23(2):140-54.

33 Chan A-W, Tetzlaff JM, Gøtzsche PC, et al. SPIRIT 2013 Explanation and Elaboration: Guidance for protocols of clinical trials. *BMJ Open*2013;346:e7586

34 Beekman AT, Deeg DJ, Van Limbeek J, et al. Brief communication.: criterion validity of the Center for Epidemiologic Studies Depression scale (CES-D): results from a community-based sample of older subjects in the Netherlands. *Psychol Med*1997;27(1):231-5.

35 Spitzer RL, Kroenke K, Williams JB, et al. A brief measure for assessing generalized anxiety disorder: the GAD-7. *Arch Intern Med*2006;166(10):1092-7.

36 Lecrubier Y, Sheehan DV, Weiller E, et al. The Mini International Neuropsychiatric Interview (MINI). A short diagnostic structured interview: reliability and validity according to the CIDI. *Eur Psychiatry*1997;12(5):224-31.

37 Weisel KK, Zarski AC, Berger T, et al. Transdiagnostic tailored internet-and mobile-based guided treatment for major depressive disorder and comorbid anxiety: study protocol of a randomized controlled trial. *Front Psychiatry*2018;9:274.

For peer review only

38 Bolinski F, Kleiboer A, Karyotaki E, et al. Effectiveness of a transdiagnostic individually tailored Internet-based and mobile-supported intervention for the indicated prevention of depression and anxiety (ICare Prevent) in Dutch college students: study protocol for a randomised controlled trial. *Trials*2018;19(1):118-30.

39 Karyotaki E, Klein AM, Riper H, et al. Examining the effectiveness of a web-based intervention for symptoms of depression and anxiety in college students: study protocol of a randomised controlled trial. *BMJ Open*2019;9(5):e028739.

40. Buntrock, C., Ebert, D. D., Lehr, D., Smit, F., Riper, H., Berking, M., & Cuijpers, P. (2016). Effect of a web-based guided self-help intervention for prevention of major depression in adults with subthreshold depression: A randomized clinical trial. *JAMA*, *315*, 1854–1863.

41 Ebert, D. D., Buntrock, C., Lehr, D., Smit, F., Riper, H., Baumeister, H., Cuijpers, P., & Berking, M. (2018). Effectiveness of web- and mobile-based treatment of subthreshold sepression with adherence-focused guidance: A single-blind randomized controlled trial. *Behavior therapy*, *49*, 71–83.

42. Ebert, D. D., Nobis, S., Lehr, D., Baumeister, H., Riper, H., Auerbach, R. P., Snoek, F., Cuijpers, P., & Berking, M. (2017). The 6-month effectiveness of Internet-based guided self-help for depression in adults with Type 1 and 2 diabetes mellitus. *Diabetic medicine : a journal of the British Diabetic Association*, *34*, 99–107.

43. Etzelmueller A, Vis C, Karyotaki E, Baumeister H, Titov N, Berking M, Cuijpers P, Riper H, Ebert D (2020). Effects of internet-based cognitive behavioral therapy in routine care for adults in treatment for depression and anxiety: Systematic Review and meta-analysis. *Journal of Medical Internet Research*, *22*;e18100

44 Kroenke K, Spitzer RL, Williams JB, et al. An ultra-brief screening scale for anxiety and depression: the PHQ–4. *Psychosomatics*2009;50(6):613-21.

For peer review only - http://bmjopen.bmj.com/site/about/guidelines.xhtml

45 Beck AT, Steer RA, Ball R, et al. Comparison of Beck Depression Inventories-IA and-II in psychiatric outpatients. *J Pers Assess*1996;67(3):588-97.

46 Kroenke K, Spitzer RL. The PHQ-9: a new depression diagnostic and severity measure. *Psychiatric Ann*2002;32(9):509-15.

47 Dear BF, Titov N, Sunderland M, et al. Psychometric comparison of the generalized anxiety disorder scale-7 and the Penn State Worry Questionnaire for measuring response during treatment of generalised anxiety disorder. *Cogn Behav Ther*2011;40(3):216-27.

48 Wittkampf KA, Naeije L, Schene AH, et al. Diagnostic accuracy of the mood module of the Patient Health Questionnaire: a systematic review. *Gen Hosp Psychiatry*2007;29(5):388-95.

49 Levis B, Benedetti A, Thombs BD. Accuracy of Patient Health Questionnaire-9 (PHQ-9) for screening to detect major depression: individual participant data meta-analysis. *BMJ Open*2019;365-75.

50 Bush K, Kivlahan DR, McDonell MB, et al. The AUDIT alcohol consumption questions (AUDIT-C): an effective brief screening test for problem drinking. *Arch Intern Med*1998;158(16):1789-95.

51 Verhoog S, Dopmeijer JM, de Jonge JM, et al. The use of the alcohol use disorders identification test–consumption as an indicator of hazardous alcohol use among university students. *Eur Addict Res*2020;26(1):1-9.

52 Skinner HA. The drug abuse screening test. *Addict Behav*1982;7(4):363-71.

53 Maisto SA, Carey MP, Carey KB, et al. Use of the AUDIT and the DAST-10 to identify alcohol and drug use disorders among adults with a severe and persistent mental illness. *Psychol Assess*2000;12(2):186-92.

54 Group TE. EuroQol-a new facility for the measurement of health-related quality of life. *Health Policy*1990;16(3):199-208.

For peer review only

55 Heslin M, Chua KC, Trevillion K, et al. Psychometric properties of the five-level EuroQoL-5 dimension and Short Form-6 dimension measures of health-related quality of life in a population of pregnant women with depression. *BJPsych Open*2019;5(6);388.

56 König HH, Heider D, Lehnert T, et al. Health status of the advanced elderly in six European countries: results from a representative survey using EQ-5D and SF-12. *Health Qual Life Outcomes*2010;8(1):143-53.

57 Matsushita M, Adachi H, Arakida M, et al. Presenteeism in college students: reliability and validity of the Presenteeism Scale for Students. *Qual Life Res*2011;20(3):439-46.

58 Bouwmans C, De Jong K, Timman R, et al. Feasibility, reliability and validity of a questionnaire on healthcare consumption and productivity loss in patients with a psychiatric disorder (TiC-P). *BMC Health Ser Res*2013;13(1):217-25.

59 Larsen DL, Attkisson CC, Hargreaves WA, et al. Assessment of client/patient satisfaction: development of a general scale. *Eval Program Plann*1979;2(3):197-207.

60 De Brey H. A cross-national validation of the client satisfaction questionnaire: the Dutch experience. *Eval Program Plann*1983;6(3-4):395-400.

61 Attkisson CC, Greenfield TK. *Client Satisfaction Questionnaire-8 and Service Satisfaction Scale-30*. In: Maruish ME, eds. *The Use of Psychological Testing for Treatment Planning and Outcome Assessment*. (pp 402–420). Lawrence Erlbaum Associates, Inc.

62 Christensen H, Griffiths KM, Farrer L. Adherence in internet interventions for anxiety and depression: systematic review. *J Med Internet Res*2009;11(2):e13.

63 Peters L, Sunderland M, Andrews G, et al. Development of a short form Social Interaction Anxiety (SIAS) and Social Phobia Scale (SPS) using nonparametric item response theory: The SIAS-6 and the SPS-6. *Psychol Assess*2012 Mar;24(1):66-76.

64 Connor KM, Kobak KA, Churchill LE, et al. Mini-SPIN: A brief screening assessment for generalized social anxiety disorder. *Depress Anxiety*2001;14(2):137-40.

For peer review only

65 Weeks JW, Spokas ME, Heimberg RG. Psychometric evaluation of the mini-social phobia inventory (Mini-SPIN) in a treatment-seeking sample. *Depress Anxiety*2007;24(6):382-91.

66 Seeley-Wait E, Abbott MJ, Rapee RM. Psychometric properties of the mini-social phobia inventory. *Prim Care Companion J Clin Psychiatry*2009;11(5):231-36.

67 Blatt SJ, D'Afflitti JP, Quinlan DM. Experiences of depression in normal young adults. *J Abnorm Psychol*1976 Aug;85(4):383.

68 Desmet M, Vanheule S, Groenvynck H, et al. The depressive experiences questionnaire. *Eur J Psychol Assess*2007;23(2):89-98.

For peer review only

Table 1. *Overview of the measures separately for each assessment point*

	Questionnaire	Assessment points					
		Baseline (t1)	Pre-sessions	Mid-treatment (t2)	Post-treatment (t3)	6 months follow-up (t4)	12 months follow-up (t5)
Anxiety	GAD-7	X	X*	X	X	X	X
Depression	PHQ-9	X	X*	X	X	X	X
Social anxiety	SIAS-6	X		X	X	X	X
Social anxiety	Mini-SPIN	X	X	X	X	X	X
Alcohol use	AUDIT-C	X		X	X	X	X
Drug use	DAST-10	X		X	X	X	X
Quality of life	EQ-5D	X			X	X	X
Objective academic achievement	**	X			X	X	X
Subjective academic achievement	PSS-WIS	X			X	X	X
Perfectionism	DEQ-SCP (short)	X					
Suicide risk	BDI-II (item 9) PHQ-4 (item 3)		X				
Medical Service Use	TiC-P				X	X	X
Satisfaction with intervention	CSQ-8				X		

For peer review only - http://bmjopen.bmj.com/site/about/guidelines.xhtml

*PHQ-4, brief anxiety and depression measure to monitor symptoms. **Self-developed questionnaire. GAD-7 = General Anxiety Disorder-7 items, PHQ-9 = Patient Health Questionnaire-9 items, SIAS-6 = Social Interaction Anxiety Scale – items, Mini-SPIN = Mini-Social Phobia Inventory, AUDIT-C = Alcohol Use Disorders Identification Test-Concise, DAST-10 = Drug Abuse Screening Test – 10 items, EQ-5D-5L = EuroQol-5D-5L, PSS-WIS = Presenteeism Scale for Students – Work Impairment Scale, DEQ-SCP = Depressive Experiences Questionnaire - Self-Critical Perfectionism, ECS-R-SF = Revised Experiences in Close Relationships – Short Form, BDI-II = Beck Depression Inventory scale –II, PHQ-4 = Patient Health Questionnaire- 4 items, TiC-P = Treatment Inventory of Costs in Patients, CSQ-8 = Client Satisfaction Questionnaire – 8 items.

For peer review only

Figure 1. Flow of the study

For peer review only

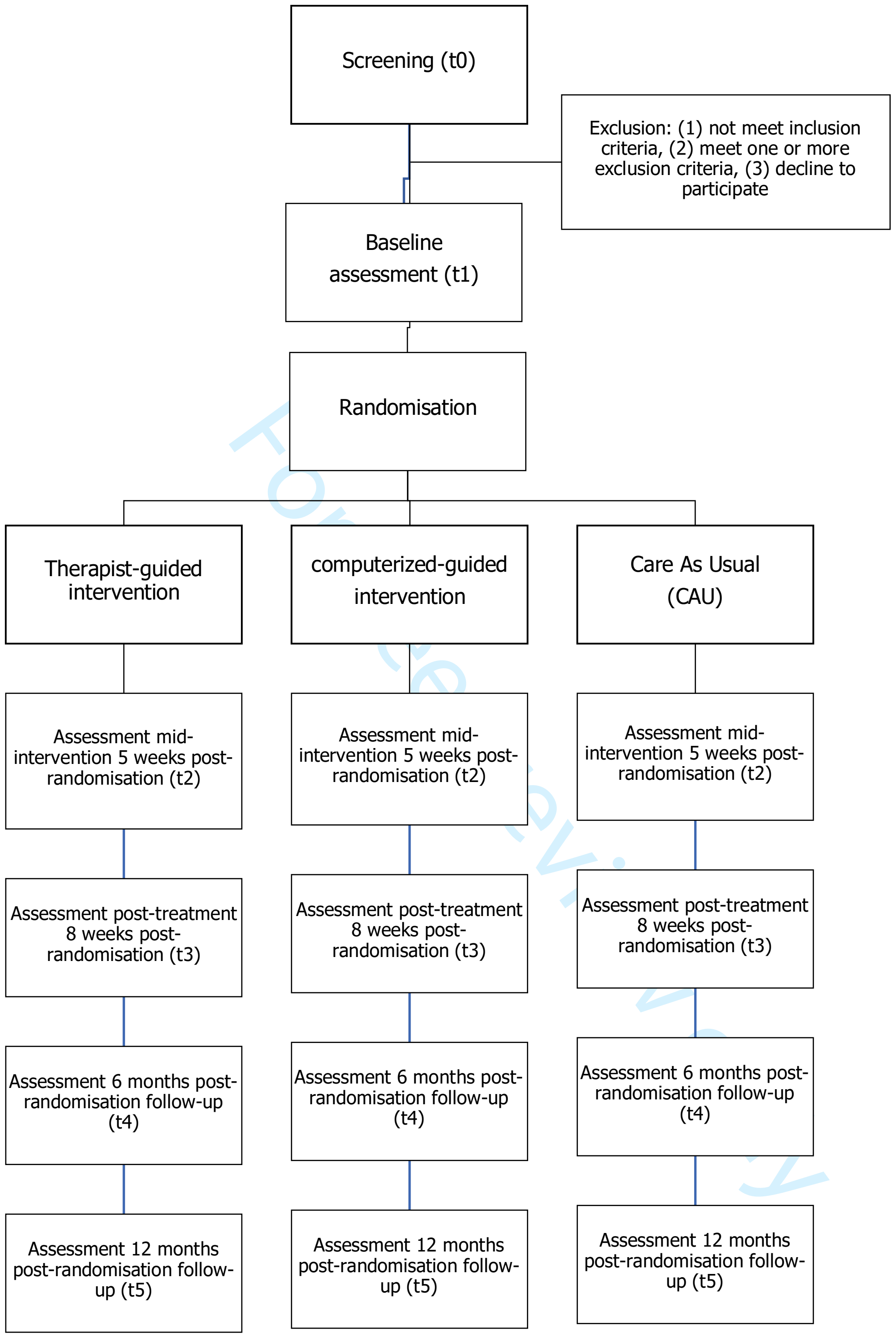
Screening (t0)
Exclusion: (1) not meet inclusion criteria, (2) meet one or more exclusion criteria, (3) decline to participate
Baseline assessment (t1)
Randomisation
Therapist-guided intervention
computerized-guided intervention
Care As Usual (CAU)
Assessment mid-intervention 5 weeks post-randomisation (t2)
Assessment mid-intervention 5 weeks post-randomisation (t2)
Assessment mid-intervention 5 weeks post-randomisation (t2)
Assessment post-treatment 8 weeks post-randomisation (t3)
Assessment post-treatment 8 weeks post-randomisation (t3)
Assessment post-treatment 8 weeks post-randomisation (t3)
Assessment 6 months post-randomisation follow-up (t4)
Assessment 6 months post-randomisation follow-up (t4)
Assessment 6 months post-randomisation follow-up (t4)
Assessment 12 months post-randomisation follow-up (t5)
Assessment 12 months post-randomisation follow-up (t5)
Assessment 12 months post-randomisation follow-up (t5)

Appendix A

Reporting checklist for protocol of a clinical trial.

Based on the SPIRIT guidelines.

Instructions to authors

Complete this checklist by entering the page numbers from your manuscript where readers will find each of the items listed below.

Your article may not currently address all the items on the checklist. Please modify your text to include the missing information. If you are certain that an item does not apply, please write "n/a" and provide a short explanation.

Upload your completed checklist as an extra file when you submit to a journal.

In your methods section, say that you used the SPIRITreporting guidelines, and cite them as:

Chan A-W, Tetzlaff JM, Gøtzsche PC, Altman DG, Mann H, Berlin J, Dickersin K, Hróbjartsson A, Schulz KF, Parulekar WR, Krleža-Jerić K, Laupacis A, Moher D. SPIRIT 2013 Explanation and Elaboration: Guidance for protocols of clinical trials. BMJ. 2013;346:e7586

		Reporting Item	Page Number
**Administrative information**			
Title	#1	Descriptive title identifying the study design, population, interventions, and, if applicable, trial acronym	1
1Trial registration	#2a	Trial identifier and registry name. If not yet registered, name of intended registry	2
Trial registration: data set	#2b	All items from the World Health Organization Trial Registration Data Set	1, 2, 8, 10, 11, 12, 18, 19, 20, 30
Protocol version	#3	Date and version identifier	header
Funding	#4	Sources and types of financial, material, and other support	19

For peer review only

Roles and responsibilities: contributorship	#5a	Names, affiliations, and roles of protocol contributors	1, 19
Roles and responsibilities: sponsor contact information	#5b	Name and contact information for the trial sponsor	19
Roles and responsibilities: sponsor and funder	#5c	Role of study sponsor and funders, if any, in study design; collection, management, analysis, and interpretation of data; writing of the report; and the decision to submit the report for publication, including whether they will have ultimate authority over any of these activities	19
Roles and responsibilities: committees	#5d	Composition, roles, and responsibilities of the coordinating centre, steering committee, endpoint adjudication committee, data management team, and other individuals or groups overseeing the trial, if applicable (see Item 21a for data monitoring committee)	18, 19
**Introduction**			
Background and rationale	#6a	Description of research question and justification for undertaking the trial, including summary of relevant studies (published and unpublished) examining benefits and harms for each intervention	5,6,7
Background and rationale: choice of comparators	#6b	Explanation for choice of comparators	5,6,7
Objectives	#7	Specific objectives or hypotheses	7
Trial design	#8	Description of trial design including type of trial (eg, parallel group, crossover, factorial, single group), allocation ratio, and framework (eg, superiority, equivalence, non-inferiority, exploratory)	8

For peer review only

**Methods: Participants, interventions, and outcomes**			
Study setting	#9	Description of study settings (eg, community clinic, academic hospital) and list of countries where data will be collected. Reference to where list of study sites can be obtained	8,9,
Eligibility criteria	#10	Inclusion and exclusion criteria for participants. If applicable, eligibility criteria for study centres and individuals who will perform the interventions (eg, surgeons, psychotherapists)	8
Interventions: description	#11a	Interventions for each group with sufficient detail to allow replication, including how and when they will be administered	10,11,12
Interventions: modifications	#11b	Criteria for discontinuing or modifying allocated interventions for a given trial participant (eg, drug dose change in response to harms, participant request, or improving / worsening disease)	8
Interventions: adherance	#11c	Strategies to improve adherence to intervention protocols, and any procedures for monitoring adherence (eg, drug tablet return; laboratory tests)	9,15,18
Interventions: concomitant care	#11d	Relevant concomitant care and interventions that are permitted or prohibited during the trial	8
Outcomes	#12	Primary, secondary, and other outcomes, including the specific measurement variable (eg, systolic blood pressure), analysis metric (eg, change from baseline, final value, time to event), method of aggregation (eg, median, proportion), and time point for each outcome. Explanation of the clinical relevance of chosen efficacy and harm outcomes is strongly recommended	13, 14, 15, 16, 28, 29

For peer review only

Participant timeline	#13	Time schedule of enrolment, interventions (including any run-ins and washouts), assessments, and visits for participants. A schematic diagram is highly recommended (see Figure)	30
Sample size	#14	Estimated number of participants needed to achieve study objectives and how it was determined, including clinical and statistical assumptions supporting any sample size calculations	16
Recruitment	#15	Strategies for achieving adequate participant enrolment to reach target sample size	18
**Methods: Assignment of interventions (for controlled trials)**			
Allocation: sequence generation	#16a	Method of generating the allocation sequence (eg, computer-generated random numbers), and list of any factors for stratification. To reduce predictability of a random sequence, details of any planned restriction (eg, blocking) should be provided in a separate document that is unavailable to those who enrol participants or assign interventions	9,10
Allocation concealment mechanism	#16b	Mechanism of implementing the allocation sequence (eg, central telephone; sequentially numbered, opaque, sealed envelopes), describing any steps to conceal the sequence until interventions are assigned	8,9,10
Allocation: implementation	#16c	Who will generate the allocation sequence, who will enrol participants, and who will assign participants to interventions	9,10
Blinding (masking)	#17a	Who will be blinded after assignment to interventions (eg, trial participants, care providers, outcome assessors, data analysts), and how	10

For peer review only

Blinding (masking): emergency unblinding	#17b	If blinded, circumstances under which unblinding is permissible, and procedure for revealing a participant's allocated intervention during the trial	n/a
**Methods: Data collection, management, and analysis**			
Data collection plan	#18a	Plans for assessment and collection of outcome, baseline, and other trial data, including any related processes to promote data quality (eg, duplicate measurements, training of assessors) and a description of study instruments (eg, questionnaires, laboratory tests) along with their reliability and validity, if known. Reference to where data collection forms can be found, if not in the protocol	11, 13,14,15,16
Data collection plan: retention	#18b	Plans to promote participant retention and complete follow-up, including list of any outcome data to be collected for participants who discontinue or deviate from intervention protocols	18
Data management	#19	Plans for data entry, coding, security, and storage, including any related processes to promote data quality (eg, double data entry; range checks for data values). Reference to where details of data management procedures can be found, if not in the protocol	19
Statistics: outcomes	#20a	Statistical methods for analysing primary and secondary outcomes. Reference to where other details of the statistical analysis plan can be found, if not in the protocol	16
Statistics: additional analyses	#20b	Methods for any additional analyses (eg, subgroup and adjusted analyses)	16

For peer review only

Statistics: analysis population and missing data	#20c	Definition of analysis population relating to protocol non-adherence (eg, as randomised analysis), and any statistical methods to handle missing data (eg, multiple imputation)	16
**Methods: Monitoring**			
Data monitoring: formal committee	#21a	Composition of data monitoring committee (DMC); summary of its role and reporting structure; statement of whether it is independent from the sponsor and competing interests; and reference to where further details about its charter can be found, if not in the protocol. Alternatively, an explanation of why a DMC is not needed	n/a
Data monitoring: interim analysis	#21b	Description of any interim analyses and stopping guidelines, including who will have access to these interim results and make the final decision to terminate the trial	18,19
Harms	#22	Plans for collecting, assessing, reporting, and managing solicited and spontaneously reported adverse events and other unintended effects of trial interventions or trial conduct	18,19
Auditing	#23	Frequency and procedures for auditing trial conduct, if any, and whether the process will be independent from investigators and the sponsor	n/a
**Ethics and dissemination**			
Research ethics approval	#24	Plans for seeking research ethics committee / institutional review board (REC / IRB) approval	18,19
Protocol amendments	#25	Plans for communicating important protocol modifications (eg, changes to eligibility criteria, outcomes, analyses) to relevant parties (eg, investigators, REC / IRBs, trial participants, trial registries, journals, regulators)	19

For peer review only

Consent or assent	#26a	Who will obtain informed consent or assent from potential trial participants or authorised surrogates, and how (see Item 32)	9
Consent or assent: ancillary studies	#26b	Additional consent provisions for collection and use of participant data and biological specimens in ancillary studies, if applicable	n/a
Confidentiality	#27	How personal information about potential and enrolled participants will be collected, shared, and maintained in order to protect confidentiality before, during, and after the trial	10, 19
Declaration of interests	#28	Financial and other competing interests for principal investigators for the overall trial and each study site	19
Data access	#29	Statement of who will have access to the final trial dataset, and disclosure of contractual agreements that limit such access for investigators	10,19
Ancillary and post trial care	#30	Provisions, if any, for ancillary and post-trial care, and for compensation to those who suffer harm from trial participation	n/a
Dissemination policy: trial results	#31a	Plans for investigators and sponsor to communicate trial results to participants, healthcare professionals, the public, and other relevant groups (eg, via publication, reporting in results databases, or other data sharing arrangements), including any publication restrictions	19
Dissemination policy: authorship	#31b	Authorship eligibility guidelines and any intended use of professional writers	20
Dissemination policy: reproducible research	#31c	Plans, if any, for granting public access to the full protocol, participant-level dataset, and statistical code	n/a

Appendices

For peer review only

Informed consent materials	#32	Model consent form and other related documentation given to participants and authorised surrogates	A
Biological specimens	#33	Plans for collection, laboratory evaluation, and storage of biological specimens for genetic or molecular analysis in the current trial and for future use in ancillary studies, if applicable	n/a

None The SPIRIT checklist is distributed under the terms of the Creative Commons Attribution License CC-BY-ND 3.0. This checklist can be completed online using https://www.goodreports.org/, a tool made by the EQUATOR Network in collaboration with Penelope.ai

For peer review only

UNIVERSITEIT VAN AMSTERDAM

Appendix B

Subject information for participation in medical scientific research

UvAcare project

Examining the effectiveness of a web-based intervention for symptoms of depression and/or anxiety among students and PhD students.

Introduction

Dear student, dear PhD student,

You are kindly asked to take part in a medical-scientific study. Participation is voluntary. Participation requires your written consent. You have received this letter because, based on the online survey that you have filled in, you appear to experience symptoms of depression and/or anxiety. This means that you might benefit from an online intervention. Before you decide whether you want to participate in this study, you will be given an explanation about what the study involves. Please read this information carefully and ask the research team for an explanation if you have any questions. You can also ask the independent expert (dhr. drs. Daan Tempelman (D.A.J.Tempelman@uva.nl, 0205254772) for additional information. You may also discuss it with your partner, classmates, friends, or family.

Additional information about participating in the study can be found in the enclosed 'Medical Research Brochure'.

1. General information

This study is conducted at the department of developmental psychology of the University of Amsterdam (UvA), in collaboration with the Student Medical Healthservice UvA-HvA and VU Amsterdam. The Medical Ethics Committee of the Academic Medical Center (AMC) has approved this study. General information about the assessment of research can be found in the 'Medical Research Brochure'.

2. Purpose of the study

First of all, the UvAcare project is part of a care project that aims to offer additional care to students and PhD students at the UvA in the coming years using different online interventions, which will be developed throughout the projectand subsequently conduct research to improve existing care and make it more accessible.

For peer review only - http://bmjopen.bmj.com/site/about/guidelines.xhtml

UNIVERSITEIT VAN AMSTERDAM

Therefore, this project will investigate the effectiveness of an online intervention for university students and PhD students with symptoms of depression and/or anxiety. The efficacy of a guided transdiagnostic intervention and an unguided, automatic, version of the same online intervention will be compared to the efficacy of treatment as usual. Treatment as usual consists of the option to seek help for example of a student psychologist, a general practitioner, or of a specialist in primary or secondary mental health care. In addition, the efficacy of the guided online intervention will be compared to the efficacy of the unguided online intervention. Principally, we will examine the reduction of depression and/or anxiety symptoms before and after treatment, and compared to no treatment at all.

3. Background of the study

The goal of the UvAcare project is to recognize mental health problems in college students and PhD students and to treat those in need at an early stage. It's important to tackle this early on because college and PhD years are considered to be a peak period for the first onset of common mental disorders, such as depression and anxiety. Preventive interventions may limit the development of symptoms and may prevent low (academic) achievement and academic drop out. Research has shown that guided web-based interventions are effective in treating symptoms of depression and anxiety in the general population. In this study we examine the effectiveness of a guided and unguided transdiagnostic treatment adapted to college students' needs. We would like to examine whether these transdiagnostic web-based interventions are effective in treating symptoms of depression and/or anxiety when compared to treatment as usual services for students and PhD students.

4. What participation involves

We kindly ask you to complete a couple of questionnaires: before the self-help online intervention, at 5 weeks, at 8 weeks, at 6 months and at 12 months. In addition we ask you to fill in a short questionnaire before each treatment session, or weekly in case of the treatment-as-usual condition (8 sessions/weeks) to monitor your mental health.

Research

If you decide that you would like to participate, you will receive an invitation for a clinical diagnostic interview administered through a phone call. The purpose of this interview is to determine whether you have a diagnosis of depression or anxiety and to check whether you are eligible to participate in this study. This interview lasts approximately 30-45 minutes. If you are eligible to participate we ask you to complete the first set of online questionnaires. After completing this, it will be determined by drawing lots, whether you will be assigned either to the guided intervention, to the unguided intervention or to treatment as usual. Your chance to be assigned to any of the groups is one third.

For peer review only - http://bmjopen.bmj.com/site/about/guidelines.xhtml

UNIVERSITEIT VAN AMSTERDAM

The online intervention

The online intervention consists of 7 sessions and one booster session 4 weeks after completing the last regular session. It is recommended to complete one session a week. One session takes about 45 to 60 minutes. The sessions consist of text, exercises and audio-visual components (e.g. videos). Participants in the unguided intervention receive automatic feedback messages and reminders, and there is a possibility for personal contact. In additon, participants in the guided intervention receive personal feedback on every session from their personal online coach.

Treatment as usual

In the treatment-as-usual condition you will receive information about existing care facilities inside and outside of the UvA. Based on this information, you will be encouraged to seek the help you might need choosing the type of care that most appeals to you.

5. What is expected of you

In order to carry out the study properly, it is important that you follow the study instructions.

The study instructions require that you:

- complete the online questionnaires at thirteen points in time: before the beginning of the online intervention, before each treatment session or weekly during the intervention (8 sessions/weeks in total), at 5 weeks, at 8 weeks, at 6 months and at 12 months after the start of the intervention,
- will be interviewed by phone four times before the beginning of the online intervention

Thus, we will contact you after the intervention for questionnaires (after the intervention, at 6 months and 12 months).

It is important that you contact the research team (uvacare@uva.nl):

- if you no longer want to participate in the study,
- if your contact details change.

6. Possible side effects/complications

We expect that participation in this study entails negligible risks.

For peer review only - http://bmjopen.bmj.com/site/about/guidelines.xhtml

UNIVERSITEIT VAN AMSTERDAM

7. Possible advantages and disadvantages

It is important that you consider the possible benefits and disadvantages before you decide to participate. An advantage of participating is the possibility of the guided or unguided online intervention. The intervention is 24 hours per day available. There is also the possibility of personal contact during this period. The intervention might have a positive effect on possible symptoms of depression and/or anxiety. Moreover, your participation may contribute to increased knowledge about the treatment and help us in determining its feasibility and efficacy for college students with symptoms of anxiety and/or depression. We expect that participation in this study does not entail any risks. However, it is possible that you may experience some levels of distress when participating in the diagnostic interview, filling out the questionnaires or working on the intervention, since elaborating on potential unpleasant emotions and working on your current problems might be confronting. In addition, participating requires some time and effort.

8. If you do not want to participate or you want to stop participating in the study

It is up to you to decide whether or not to participate in the study. Participation is voluntary. If you participate in the study, you can always change your mind and decide to stop, at any time during the study. Moreover, you do not have to say why you are stopping. The data collected until that time will still be used for the study. If there is any new information about the study that is important to you, the investigator will let you know. You will then be asked whether you still want to continue your participation.

9. End of the study

Your participation in the study stops when:

- You have completed all the questionnaires
- You choose to stop
- The investigator considers it best for you to stop
- The government or Medical Research Ethics Committee decides to stop the study.

The study is concluded once all the participants have completed all the questionnaires of the study.

After processing the data, the investigator will inform you about the most important results of the study.

For peer review only - http://bmjopen.bmj.com/site/about/guidelines.xhtml

UNIVERSITEIT VAN AMSTERDAM

10. Usage and storage of your data

Your personal data will be collected, used and stored for this study. This concerns data such as your name, address, date of birth and data about your health. The collection, use and storage of your data is required to answer the questions asked in this study and to publish the results. Your data will also be forwarded to the VU Amsterdam, governed by the same European guidelines for personal data protection. This, however, includes solely data that are essential for the study. We ask your permission for the use of your data.

Confidentiality of your data

To protect your privacy, any personal information that could identify you will be stored separately from your research data and will be given a code. Your name and other information that can directly identify you will be omitted. Data can only be traced back to you with the encryption key. The encryption key remains safely stored in the local research institute. The data that is sent to the sponsor will only contain the code, not your name or other data with which you can be identified. The data cannot be traced back to you in reports and publications about the study.

Access to your data for verification

Some people can access all your data at the research location. Including the data without a code. This is necessary to check whether the study is being conducted in a good and reliable manner. Persons who have access to your data for review are members of the research team who monitor the study, the principal investigators, and national and international supervisory authorities, for example, the Healthcare and Youth Inspectorate. They will keep your data confidential. The members of the research team will only trace back to an individual in the case of an emergency, e.g. suicidal ideation. In that case the research team will contact the participant and provide information on further help. It is therefore possible that the research team will contact you during the intervention. However, this is only the case if you consider hurting yourself and seem to be in need of extra help. We ask you to consent to this access.

Retention period of your data

Your data must be kept for 15 years at the research location.

Storage and use of data for other research

Your data may also be of importance for other scientific research in the field of web-based intervention in treating symptoms of depression and/or anxiety. To this end, your data will be stored for 15 years. You can indicate on the consent form whether or not you agree with this. If you do not agree with this, you can still participate in the current study.

Withdrawing consent

You can withdraw your consent to the use of your personal data at any time. This applies to this study and also to storage and use for future research. The study data collected until the moment you withdraw your consent will still be used in the study.

For peer review only - http://bmjopen.bmj.com/site/about/guidelines.xhtml

For peer review only

UNIVERSITEIT VAN AMSTERDAM

More information about your rights when processing data

For general information about your rights when processing your personal data, you can consult the website of the Dutch Data Protection Authority.

If you have questions about your rights, please contact the person responsible for the processing of your personal data. For this study, that is:

Dhr. Prof Dr. Reinout Wiers, University of Amsterdam. See Appendix A for contact details.

If you have questions or complaints about the processing of your personal data, we advise you to first contact the research location. You can also contact the Data Protection Officer of the UvA (please see contact details in Appendix A) or the Dutch Data Protection Authority.

Registration of the study

Information about this study is included in a list of medical-scientific studies namely CCMO [NL64929.018.18/ToetsingOnline]. It does not contain any information that can be traced to you. After the study, the website may display a summary of the results of this study.

11. Compensation for participation

The internet-based intervention used in the study is free of charge to you. Participants who will complete the follow-up assessments will be offered a voucher worth of 10 euros. Additionally, amongst every 100 of these students, a tablet will be granted. If you stop before the study is over, you will not receive a compensation for participation.

12. Any questions?

If you have any questions, please contact the **study team** through email or phone (uvacare @uva.nl, secretary 020-5256830). If you would like any independent advice about participation in this study, you may contact dhr. drs. Daan Tempelman (D.A.J.Tempelman@uva.nl, 020-5254772). He knows about the study but is not involved in it.

If you have any complaints, you may contact the member of the Ethics Review Board (ERB) of the Faculty of Social and Behavioural Sciences (FMG) of the University of Amsterdam: dhr. dr. Wery van den Wildenberg, W.P.M.vandenWildenberg@uva.nl, 020-5256686. All the relevant details can be found in **Appendix A**: Contact details.

For peer review only - http://bmjopen.bmj.com/site/about/guidelines.xhtml

UNIVERSITEIT VAN AMSTERDAM

13. Signing the consent form

If you've had enough time to consider and you would like to participate in the study, we will ask you to confirm this in writing on the appended consent form. By your written permission you indicate that you have understood the information and consent to participation in the study. There are three ways to give your written consent:

- Print the consent form, check all relevant boxes, sign the form and send it via post to the following address (no stamp required):

  UvA-FMG
  Ontwikkelingspsychologie
  t.a.v. UvAcare (code 1041)
  Antwoordnummer 3521
  1000 RA Amsterdam

- If you're not able to print and/or send the consent form you can use the link in the invitation e-mail to leave your address details. We will send an envelope with a printed form and a retour envelope. You can easily sign your consent and return the form via post.
- If you rather sign and give the form directly to us there are informed consent forms at the Secretaries Office of Developmental Psychology, that you can sign directly.

Any questions about the consent form will be answered by the research team. The signature sheet is kept by the researcher.

Thank you for your attention.

Kind regards,

Mr. Dr. R. Wiers, professor Developmental Psychology
Ms. Dr. A. Klein, Senior Researcher Developmental Psychology
Mr. P. Vonk, director General Practitioners Practice UvA-HvA
Ms. C. van der Heijde, Senior Researcher General Practitioners Practice UvA-HvA

14. Appendices to this information

A. Contact details
B. Informed Consent Form
C. 'Medical Research Brochure: General information for Subjects'

For peer review only - http://bmjopen.bmj.com/site/about/guidelines.xhtml

UNIVERSITEIT VAN AMSTERDAM

Appendix A: contact details

Principal Investigators:

Mr. Prof. Reinout Wiers,
Professor Developmental Psychology

Ms. Dr. Anke Klein,
Senior Researcher Developmental Psychology
University of Amsterdam, Faculty of Behavioural and Movement Sciences
Programme group Developmental Psychology
Postbus 15916, 1001 NK Amsterdam

Secretary: Mrs. Helma Kruijk | E: W.G.S.M.Kruijk@uva.nl | T: 0205256830

Mr. Peter Vonk, MD,
Director General Practitioners Practice UvA-HvA,

Ms. Dr. Claudia van der Heijde,
Senior Researcher General Practitioners Practice UvA-HvA
University of Amsterdam,
Department of Research, development and prevention
Oude Turfmarkt 151, 1012 GC Amsterdam

Secretary: Mrs. Ester van Run | E: E.M.M.vanRun@uva.nl | T: 0205252857

Research team email: uvacare@uva.nl

Independent expert:

Dhr. Drs. Daan Tempelman
General practitioner,
Student Medical Healthservice UvA-HvA
University of Amsterdam
Oude Turfmarkt 151
1012 GC Amsterdam
E: D.A.J.Tempelman@uva.nl | T: 0205254772

Complaints contact:

Dhr. Dr. Wery van den Wildenberg
Faculty of Behavioural and Movement Sciences
Programme group Developmental Psychology
Postbus 15916, 1001 NK Amsterdam
E: W.P.M.vandenWildenberg@uva.nl | T: 0205256686

For peer review only

For peer review only - http://bmjopen.bmj.com/site/about/guidelines.xhtml

Data Protection Officer:
Mw. mr. drs. J.M.C. Krol
Spui 21, Kamernummer: 1.18
1012 WX Amsterdam E:
fg@uva.nl

For peer review only

For peer review only - http://bmjopen.bmj.com/site/about/guidelines.xhtml

UNIVERSITEIT VAN AMSTERDAM

Appendix B: Informed consent participant

UvAcare project

Title: *Examining the effectiveness of a web-based intervention for symptoms of depression and/or anxiety in university students and PhD students.*

- I have read the subject information form. I was also able to ask questions. My questions have been answered to my satisfaction. I had enough time to decide whether to participate.
- I know that participation is voluntary. I know that I may decide at any time not to participate after all or to withdraw from the study. I do not need to give a reason for this.
- I know that some people may have access to all my data to verify the study. These people are listed in this information sheet. I consent to the inspection by them.
- I can give consent at the start of the telephonic diagnostic interviews to record the phone call. If I do not agree with this, I can still participate in this study.
- I agree to my data being forwarded to other universities within the European Union, governed by European guidelines for personal data protection. This must, however, be essential for the study. The data must be shared in encoded form without stating my name.
- I **do** ☐
  do no☐
  consent to keeping my personal data longer and to use it for future research in the field of web- based intervention in treating symptoms of depression and/or anxiety.
- I **do** ☐
  do no☐
  consent to being contacted again after this study for a follow-up study
- I want to participate in this study.

Name participant:
Email address participant:
Signature: Date : ___/___/ _____

I hereby declare that I have fully informed this study subject about this study.

If information comes to light during the course of the study that could affect the study subject's consent, I will inform him/her of this in a timely fashion.

Name of investigator (or his/her representative):
Signature: Date: / / _

For peer review only - http://bmjopen.bmj.com/site/about/guidelines.xhtml
For peer review only

Appendix C

Example of automated feedback in the computer-guided condition:

After completing for example session six, all participants receive the same message in the messaging function; "*Dear participant, Congratulations on finishing the sixth session. We know the training is not always easy, so well done. In this session you were once again reminded that practice makes perfect. Keep trying to find the motivation you had when you started and use the tips from all the sessions thus far to work on difficult situations and challenges. You are now starting with the second to last session of the training module. We wish you once again good luck with the next session! Kind regards, the research team*".

Example of feedback in the therapist-guided condition:

An example of feedback on the sixth session; "*Dear [name of participant], Thank you for sending in the exercises from the sixth and second-to-last session. It's great that you have been persistent with this training over the past weeks! You started this session by taking stock of how your challenges from last week went. It is nice to read that you have faced your first challenge. You have taken a first step to actively conquer your fears, well done! Have you thought about rewarding yourself? This week you detected a safety behavior: carrying items to prepare for the worst-case scenarios despite never having to use them. It's great you could identify it, since you can only start making changes once you have become aware of these behaviours! In this session you have chosen to stick with the challenging area you picked last time, which was dealing with situations where there is no clear process like a queue in a busy cafe. It's great that you made a plan to challenge yourself in the coming week! You might benefit from making sure the link is clear between the activities you planned and the challenging area you chose to focus on. The more concrete and specific your plan is, the*

For peer review only

easier it will be to carry out. It is also important to have a detailed plan so you can accurately judge the difficulty of each situation. This way, you can start with easier challenges and build your way up to harder ones. To follow the examples from the previous session, your plan could be something like "Monday: order coffee with a friend (easier), Wednesday: go alone to the doctor's office where there is a clear system for the queue (moderate), Saturday: order coffee on my own (harder)". It is also possible to start with very small tasks: sometimes just imagining the situation can be a useful first step. Don't forget that every attempt is worth being rewarded and deserves respect! Plan something nice or fun for yourself as soon as you have confronted a situation. This week you also completed the optional module on rest and relaxation. I hope these strategies were useful for you and that you can use them going forward. I am also glad to see that you are continuing to plan positive activities to incorporate into your daily life, like going for a walk, getting takeout, or visiting your friends! In the seventh (and for now last) session of this training you will have the opportunity to draw up a plan for the future. You will get to plan exercises for the coming weeks, and we will look back on the training and your successes. I am looking forward to hearing back from you then! Kind regards, [name of coach]".

For peer review only
